# Ultrasensitive Near‐Infrared Circularly Polarized Light Detection Using 3D Perovskite Embedded with Chiral Plasmonic Nanoparticles

**DOI:** 10.1002/advs.202104598

**Published:** 2022-01-02

**Authors:** Hongki Kim, Ryeong Myeong Kim, Seok Daniel Namgung, Nam Heon Cho, Jung Bae Son, Kijoon Bang, Mansoo Choi, Seong Keun Kim, Ki Tae Nam, Jong Woo Lee, Joon Hak Oh

**Affiliations:** ^1^ School of Chemical and Biological Engineering Institute of Chemical Processes Seoul National University 1 Gwanak‐ro, Gwanak‐gu Seoul 08826 Republic of Korea; ^2^ Department of Chemical Engineering Pohang University of Science and Technology (POSTECH) Pohang 37673 Republic of Korea; ^3^ Department of Chemistry Myongji University 116 Myongji‐ro Yongin Gyeonggi‐do 17058 Republic of Korea; ^4^ Department of Materials Science and Engineering Seoul National University 1 Gwanak‐ro, Gwanak‐gu Seoul 08826 Republic of Korea; ^5^ Department of Chemistry Seoul National University 1 Gwanak‐ro, Gwanak‐gu Seoul 08826 Republic of Korea; ^6^ Department of Mechanical and Aerospace Engineering Seoul National University 1 Gwanak‐ro, Gwanak‐gu Seoul 08826 Republic of Korea; ^7^ Global Frontier Center for Multiscale Energy Systems Seoul National University 1 Gwanak‐ro, Gwanak‐gu Seoul 08826 Republic of Korea

**Keywords:** 3D perovskites, chiral plasmonic nanoparticles, circularly polarized light, mixed Pb—Sn perovskites

## Abstract

Chiral organic ligand‐incorporated low‐dimensional metal‐halide perovskites have received increasing attention for next‐generation photodetectors because of the direct detection capability of circularly polarized light (CPL), which overcomes the requirement for subsidiary optical components in conventional CPL photodetectors. However, most chiral perovskites have been based on low‐dimensional structures that confine chiroptical responses to the ultraviolet (UV) or short‐wavelength visible region and limit photocurrent due to their wide bandgap and poor charge transport. Here, chiroptical properties of 3D Cs_0.05_FA_0.5_MA_0.45_Pb_0.5_Sn_0.5_I_3_ polycrystalline films are achieved by incorporating chiral plasmonic gold nanoparticles (AuNPs) into the mixed Pb—Sn perovskite, without sacrificing its original optoelectronic properties. CPL detectors fabricated using chiral AuNP‐embedded perovskite films can operate without external power input; they exhibit remarkable chirality in the near‐infrared (NIR) region with a high anisotropy factor of responsivity (*g*_res_) of 0.55, via giant plasmon resonance shift of chiral plasmonic AuNPs. In addition, a CPL detector array fabricated on a plastic substrate demonstrates highly sensitive self‐powered NIR detection with superior flexibility and durability.

## Graphical abstract

Highly sensitive self‐powered near‐infrared circularly polarized light (NIR CPL) detectors are realized using 3D mixed Pb—Sn perovskite embedded with chiral plasmonic gold nanoparticles. The resulting CPL detectors exhibit remarkable discrimination ability for NIR CPL with a high *g*_res_ of 0.55 under zero‐bias, the highest value in comparison with chiral 2D perovskite‐based CPL detectors.

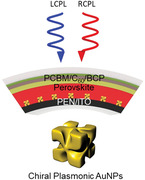

## 1 Introduction

Chiral materials that are distinguishable from their mirror images typically exhibit nonlinear optical responses to circularly polarized light (CPL), where the electric vector with a constant magnitude rotates in a plane perpendicular to the propagation direction with a steady angular velocity. The incident left‐handed circularly polarized light (LCPL) or right‐handed circularly polarized light (RCPL) can induce differentiated circulation of electronic charges (i.e., helical redistribution) in chiral materials, resulting in different degrees of light absorption.^[^ ^1^, ^2^, ^3^ ^]^ Because this function reflects intrinsic opto‐physical and chemical information regarding materials,^[^ ^4^, ^5^ ^]^ directly distinguishing between the two polarizations of CPL without subsidiary optical components (e.g., linear polarizer and quarter‐wave plate) enables miniaturization of the integrated device at a low cost and has considerable potential in many applications, including quantum optics,^[^ ^6^ ^]^ encrypted optical communication,^[^ ^7^ ^]^ spintronics,^[^ ^8^ ^]^ security surveillance,^[^ ^9^ ^]^ and remote sensing.^[^ ^10^ ^]^ In nature, stomatopod crustaceans (e.g., mantis shrimps, cuttlefish, and octopus) can discriminate the circular polarization of light; the acquired information assists with predation, navigation, and intraspecific communication within various ecosystems.^[^ ^11^ ^]^ Conventional Si or GaAs‐based CPL detectors equipped with a linear polarizer and quarter‐wave plate cause significant loss of sensitivity in light‐sensing systems. Hence, the development of photodetectors capable of directly detecting CPL has been an innovative target for advanced sensing systems.

Metal halide perovskites with a distinct 3D ABX_3_ (A = Cs, CH_3_NH_3_ or NH_2_CHNH_2_; B = Pb, Sn; and X = I, Br, Cl) crystal structure have been extensively explored in optoelectronic devices because of their outstanding optoelectronic properties, which include a high absorption coefficient,^[^ ^12^ ^]^ long diffusion length,^[^ ^13^ ^]^ and low defect density.^[^ ^14^ ^]^ Notably, the structural flexibility in low‐dimensional perovskites imparts chirality to the perovskite by inserting bulky chiral organic molecules into the perovskite lattices.^[^ ^15^ ^]^ The chiroptical effects of perovskites can be realized by the transfer of chirality from the chiral molecule to the inorganic sublattice.^[^ ^16^ ^]^ Prominent CPL detectors have been based on chiral 2D perovskites using chiral organic spacers with a pair of enantiomers such as *α*‐phenylethylamine (*α*‐PEA),^[^ ^17^, ^18^, ^19^ ^]^ *β*‐methylphenethylamine (*β*‐MPA),^[^ ^20^, ^21^ ^]^ 1‐(1‐naphthyl)ethylamine (NEA),^[^ ^22^ ^]^ and 1‐(4‐bromophenyl)ethylammonium (BPEA).^[^ ^23^ ^]^ The direct detection of CPL enabled by chiral 2D perovskites has created great interest in the chiral photonics community. However, most perovskites used for CPL detectors have been confined to 2D perovskites, which exhibit optoelectronic properties that conflict with requirements for excellent charge transfer properties in out‐of‐plane directions and broad absorption bands. Practically, typical chiral 2D perovskites exhibit no chiroptical responses in the wavelength range from visible red to near‐infrared (NIR). Very recently, the chiroptical response in the NIR region of 2D perovskites using a chiral ligand that strongly induces a two‐photon absorption was reported,^[^ ^23^ ^]^ but only limited discrimination ability for NIR CPL detection was achieved due to the inherently low absorption in the NIR region of 2D perovskite. Moreover, typical chiral 2D perovskites without crystallographic orientation engineering for out‐of‐plane charge extraction show much lower incident photon‐to‐current conversion efficiency than do their 3D counterparts, thus producing several orders of magnitude lower photocurrents. These factors have significantly impeded further breakthroughs for the development of advanced CPL detectors using perovskites. Therefore, we aimed to fabricate a CPL detector that can use 3D perovskites without compromising superior charge transport in the out‐of‐plane direction and with wide absorption bands.

The introduction of chiral plasmonic metamaterials (e.g., chiral plasmonic nanostructures or nanoparticles) into 3D perovskites is a promising option. Over the past decade, advances in chiral plasmonics have provided new insights for creating artificial chiral media or nanostructures that promote strong light‐matter interactions in the plasmonic resonant regions, where the strongly induced chirality can be superior to chiral organic molecules (e.g., helicene). Combined systems consisting of chiral plasmonic metasurfaces and inorganic semiconductors were rarely used to fabricate CPL detectors,^[^ ^24^, ^25^ ^]^ where the chiral plasmonic metasurfaces were fabricated by top‐down approaches. These results suggest the feasibility of fabricating CPL detectors using an integrated platform, chiral plasmonic elements in conjunction with achiral materials. However, the direct detection of CPL using a system that combines a chiral plasmonic element with achiral 3D perovskites has not been reported thus far. Strong localized surface plasmon resonance (LSPR) can be induced from the electron‐magnetic interaction of the metal (e.g., Au, Ag, and Cu) nanoparticles with incident light of a specific wavelength, which results in strongly enhanced electromagnetic fields and plasmon‐enhanced absorption.^[^ ^26^ ^]^ Due to this function, plasmonic nanoparticles have been employed in optoelectronic applications to improve the performance.^[^ ^27^ ^]^ Practically, the incorporation of plasmonic nanoparticles into active layers in perovskite optoelectronic devices can significantly enhance light absorption and device performance,^[^ ^28^, ^29^, ^30^ ^]^ and an integrated system composed of chiral plasmonic nanoparticles and perovskites may facilitate the development of highly efficient CPL detectors.

Here we report the development of high‐performance CPL detectors using 3D perovskites by embedding chiral plasmonic gold nanoparticles (AuNPs) into low bandgap 3D mixed Pb—Sn perovskites with a typical stoichiometry of Cs_0.05_FA_0.5_MA_0.45_Pb_0.5_Sn_0.5_I_3_. Considering that the chiral plasmonic metasurface fabricated from the top‐down method, which has been previously used in conjunction with achiral materials, requires a complicated etching process and high fabrication cost, our strategy using the chiral plasmonic AuNPs synthesized by the simple bottom‐up method can provide a distinctively effective design rule for fabricating CPL detectors. In addition, compared with conventional low‐dimensional perovskites that have been intercalated with chiral organic ligands, the developed hybrid system of chiral plasmonic AuNPs and 3D perovskites imparted chirality without compromising the outstanding optoelectronic properties of 3D perovskites. Notably, the plasmonic resonance of AuNPs could be tuned by controlling the surrounding materials, presumably because of the resonance shift by the refractive index of the surrounding medium. Spectral matching between the plasmonic resonance and the absorption of perovskites enabled distinct discrimination of CPL in the visible red and NIR wavelength regions. The outstanding anisotropy factor of responsivity (*g*_res_ > 0.5) was achieved in a CPL detector under NIR CPL illumination, the highest *g*_res_ compared with chiral 2D perovskite‐based CPL detectors.^[^ ^17^, ^18^, ^19^, ^20^, ^21^, ^23^ ^]^ Moreover, we designed a simplified vertical device configuration without a hole‐transporting layer (HTL) using mixed Pb—Sn perovskites. This device allowed efficient photovoltaic collection of charges that can be operated under zero‐bias, distinct from conventional lead‐based counterparts that require an external bias for direct detection of CPL. Finally, a self‐powered flexible CPL detector array was successfully implemented to display NIR CPL mapping. Our results are of great importance because this novel strategy will contribute to the identification of effective strategies for CPL detection solely using achiral 3D perovskites; it does not compromise the excellent optoelectronic properties of traditional 3D perovskites.

## 2 Results and Discussion

### 2.1 Chiral Plasmonic AuNP‐Embedded Perovskites

**Figure 1:**
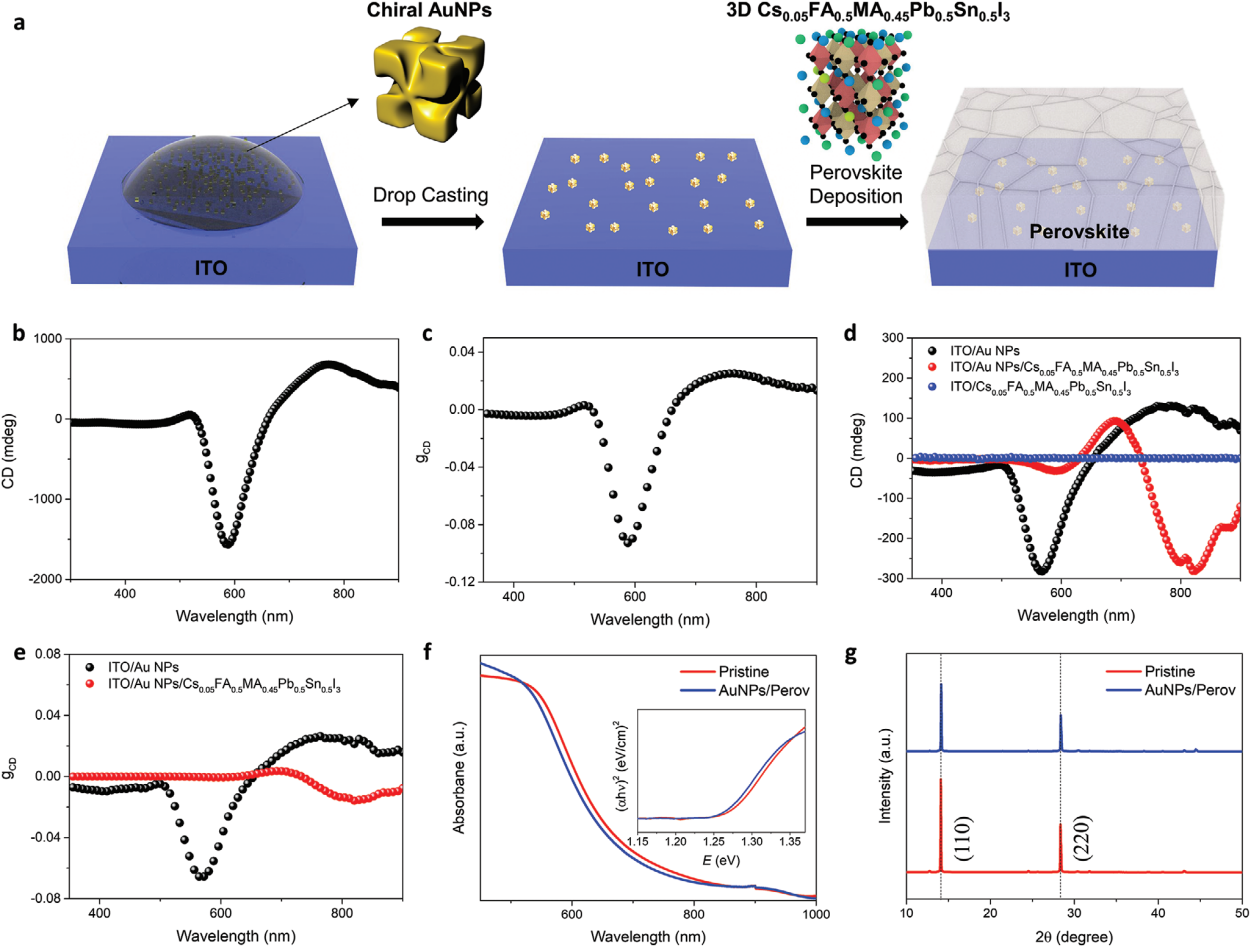
Chiral Plasmonic AuNP‐embedded perovskites. a) Schematic fabrication procedures for chiral plasmonic AuNP‐embedded perovskites. b) CD and c) *g*_CD_ spectra of AuNPs dispersed in an aqueous solution. d) CD spectra on ITO/AuNPs, ITO/AuNPs/Cs_0.05_FA_0.5_MA_0.45_Pb_0.5_Sn_0.5_I_3_, ITO/ Cs_0.05_FA_0.5_MA_0.45_Pb_0.5_Sn_0.5_I_3_ films, and e) their corresponding *g*_CD_. f) Absorption spectra of ITO/Cs_0.05_FA_0.5_MA_0.45_Pb_0.5_Sn_0.5_I_3_ (pristine) and ITO/AuNPs/ Cs_0.05_FA_0.5_MA_0.45_Pb_0.5_Sn_0.5_I_3_ films. Tauc plots in inset illustrate that the bandgap is not affected by embedded AuNPs. g) X‐ray diffraction patterns of ITO/Cs_0.05_FA_0.5_MA_0.45_Pb_0.5_Sn_0.5_I_3_ (pristine) and ITO/AuNPs/ Cs_0.05_FA_0.5_MA_0.45_Pb_0.5_Sn_0.5_I_3_ films.

The synthesis of chiral plasmonic AuNPs was conducted as described in a previous report (see Experimental Section for detailed experimental procedures),^[^ ^31^ ^]^ where the amino acid and peptide‐directed asymmetric evolution of the nanoparticles was induced to form helicoid morphologies. The asymmetric evolution of AuNPs with helicoid morphologies can endow strong chiroptical responses as discussed in previous reports.^[^ ^31^, ^32^ ^]^ The synthesized chiral plasmonic AuNPs were dispersed in an aqueous hexadecyltrimethylammonium bromide (CTAB) solution, which can stabilize [AuX_4_]^−^ (X = halide anion) complexes. The procedure to fabricate an embedded configuration of AuNPs in perovskite for device applications is illustrated in **Figure** **1**a. The aggregation of AuNPs on indium tin oxide (ITO) glass was suppressed during drying under ambient conditions by means of drop‐casting on a hot plate at a mild heat of 40 °C under an upside‐down petri dish. In this manner, we obtained uniformly distributed AuNPs on ITO; otherwise, the evaporation of droplets containing dispersed nanoparticles under ambient conditions formed a highly aggregated ring‐like pattern of AuNPs (Figure S1, Supporting Information), which can be explained by the coffee‐ring effect.^[^ ^33^, ^34^ ^]^ After preparation of the ITO/AuNP substrate, the perovskite film was spin‐coated on the substrate, leading to chiral plasmonic AuNP‐embedded perovskite films. The compact perovskite film without pinholes on the ITO/AuNPs substrate was verified using scanning electron microscopy (SEM) analysis (Figure S2, Supporting Information). Figure 1b,c displays the circular dichroism (CD) spectra of chiral AuNPs in an aqueous solution and the corresponding anisotropy factor of CD (*g*_CD_) at a plasmon resonance wavelength of approximately 600 nm. The spectra originated from the non‐perpendicular orientation between magnetic and electric dipole moments, caused by the highly twisted chiral structures of AuNPs.^[^ ^31^ ^]^ The chiroptical response of chiral AuNPs in an aqueous solution was maintained after drop‐casting the solution on an ITO substrate with a slightly blue‐shifted resonance wavelength. Meanwhile, the intensity of the CD signal was an order of magnitude lower for ITO/chiral AuNPs than for the solution, which is attributed to the low density of AuNPs during light propagation. Notably, a significant resonance shift toward the higher wavelength was observed with the perovskite film deposited on ITO/chiral AuNPs with an analogous CD intensity, as shown in Figure 1d. In contrast, the perovskite film deposited on bare ITO showed no CD signals as expected. A lower *g*_CD_ than ITO/AuNPs, but a distinct *g*_CD_ of approximately 0.02 at the NIR region, appeared with the ITO/AuNP/perovskite film (Figure 1e); this lower *g*_CD_ is attributed to the high absorption of the perovskite film. This result confirms the chiroptical response in 3D perovskites related to the presence of chiral plasmonic AuNPs, without sacrificing the optoelectronic and crystallographic properties of the original 3D Cs_0.05_FA_0.5_MA_0.45_Pb_0.5_Sn_0.5_I_3_ as shown in Figure 1f,g.

### 2.2 Plasmonic Resonance Shift of Chiral AuNPs by Surrounding Media

**Figure 2:**
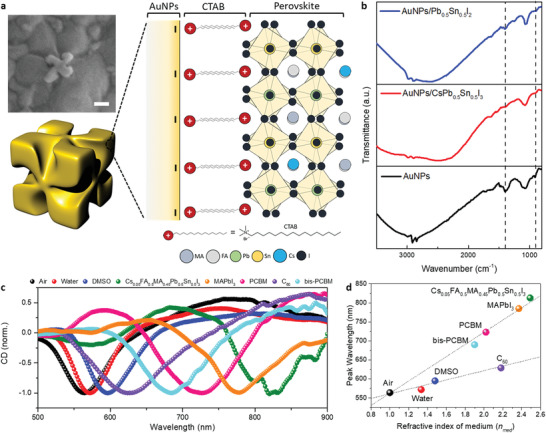
Chiroptical responses of chiral plasmonic AuNPs. a) Schematic illustration of possible interactions between AuNPs and Cs_0.05_FA_0.5_MA_0.45_Pb_0.5_Sn_0.5_I_3_. Surface SEM micrograph shows the morphology of perovskite film prepared separately by a mixed solution of perovskite precursor and AuNPs. Scale bar indicates 100 nm. b) FT‐IR spectra of ITO/AuNPs, ITO/AuNPs/Pb_0.5_Sn_0.5_I_2_, and ITO/AuNPs/ CsPb_0.5_Sn_0.5_I_3_ films. c) CD spectra of AuNPs with various surrounding media. d) *n*_med_ (at the wavelength of 580 nm)‐dependent *λ*_res_ in CD spectra, where *n* of MAPbI_3_ was obtained from the previous result.^[^ ^44^ ^]^

The LSPR effect in AuNPs caused by light with a larger wavelength than AuNPs induces collective oscillations, which reaches a plasmonic resonance at a specific wavelength depending on nanoparticles size,^[^ ^35^ ^]^ distance between nanoparticles,^[^ ^36^ ^]^ material constitution,^[^ ^37^ ^]^ geometry,^[^ ^38^ ^]^ and the surroundings of the nanoparticles.^[^ ^39^ ^]^ The main peak on the CD spectrum of pristine AuNPs was shifted when they were covered with a Cs_0.05_FA_0.5_MA_0.45_Pb_0.5_Sn_0.5_I_3_ film. This result suggests that the surrounding Cs_0.05_FA_0.5_MA_0.45_Pb_0.5_Sn_0.5_I_3_ might affect the plasmonic resonance of AuNPs. Morphological compactness between AuNPs and perovskite may ensure the behavior of the perovskite as an isotropic surrounding medium that can affect the resonance wavelength of AuNPs as shown in SEM images in **Figure** **2**a and Figure S3 in the Supporting Information. The schematic illustration in Figure 2a shows the possible interaction between AuNPs and perovskite, where quaternary ammonium groups of CTAB that cap AuNPs may be intercalated into the A‐site in perovskite instead of the MA, FA, or Cs cations. This was confirmed by Fourier‐transform infrared (FT‐IR) spectroscopy. The appearance of symmetric or asymmetric C—H scissoring vibrations of the H_3_C—N^+^ moiety (approximately 1450 cm^−1^) and C—N^+^ stretching (approximately 960 cm^−1^) in the ITO/AuNPs sample can be attributed to the CTAB capping AuNPs (Figure 2b, bottom).^[^ ^40^ ^]^ After the deposition of a thin inorganic sublattice of Pb_0.5_Sn_0.5_I_2_ and CsPb_0.5_Sn_0.5_I_3_ on the ITO/AuNPs substrate, partial (Figure 2b, top) and complete (Figure 2b, middle) disappearance of those peaks were observed, respectively, where CsPb_0.5_Sn_0.5_I_3_ was used instead of Cs_0.05_FA_0.5_MA_0.45_Pb_0.5_Sn_0.5_I_3_ for FT‐IR analysis to exclude the complicated interpretation by FA or MA. These results show that the ammonium moiety of CTAB is bound to the perovskite, implying possible interactions between the AuNPs and the perovskite. Importantly, both ITO/AuNPs/Pb_0.5_Sn_0.5_I_2_ and ITO/AuNPs/CsPb_0.5_Sn_0.5_I_3_ films showed main peak shifts toward the longer wavelength in CD spectra, compared with ITO/AuNPs (Figure S4, Supporting Information). To speculate on the origin of the shift in the CD spectra based on the surrounding media of AuNPs, we characterized the chiroptical responses of AuNPs with various surrounding media (Figure S5, Supporting Information). Normalized CD spectra with various surrounding media are shown in Figure 2c, where the degree of red‐shift showed differences according to the surrounding media.

In plasmonics, the origin of the plasmon resonance shift with surrounding media can be represented by an electrostatic model when the wavelength is much larger than the particle dimensions. The induced polarizability *α* by a dipole moment inside the particle can be defined as follows,^[^ ^41^ ^]^

(1) $$\alpha \;=\;4\pi a^{3}\frac{\varepsilon -\varepsilon_{\mathrm{med}}}{\varepsilon +2\varepsilon_{\mathrm{med}}}$$

where *a* is the dimension of the particle, *ε* is the complex dielectric function of the particle, and *ε*_med_ is the complex dielectric function of the surrounding medium. The polarizability experiences a resonance enhancement when |*ε* + 2*ε*_med_| is at a minimum, which can be simplified to the following condition for small or slowly varying Im[*ε*] (imaginary part of the dielectric function of the particle),

(2) $$Re\left[\varepsilon \left(\omega \right)\right]\;=\;-2\varepsilon_{\mathrm{med}}$$

where Re[*ε*(*ω*)] is the real part of the dielectric function of the particle and is expressed by the equation: 1−*ω*_p_^2^/*ω* ^2^; here, *ω*_p_ is the plasma frequency of the free electron gas and *ω* is the resonance frequency of the localized surface plasmon in the particle. This illustrates that the resonance red‐shifts as *ε*_m_ increases. Because the refractive index of the media (*n*_med_) is related to the dielectric constant via *n*_med_^2^ = *ε*_med_, a concomitant red‐shift of plasmonic resonance wavelength with higher *n*_med_ has been revealed and explored for optical sensing using a linear relationship of resonance wavelength to *n*_med_.^[^ ^42^, ^43^ ^]^

From this perspective, Figure 2d shows resonance peak wavelengths (*λ*_res_) at CD spectra depending on *n*_med_, where the *n* of each solid surrounding medium was extracted with a single layer of each material from ellipsometry spectroscopy (Figure S6, Supporting Information) and the *n* of MAPbI_3_ was obtained from the previous result.^[^ ^44^ ^]^ A consistent linear relationship of *λ*_res_ to *n*_med_ was observed with surrounding media of air, water, dimethyl sulfoxide (DMSO), and C_60_. Furthermore, the linear dependence of *λ*_res_ on *n*_med_ with a higher slope was observed with the surrounding media of phenyl‐C_61_‐butyric acid methyl ester (PCBM), PCBM bisadduct (bis‐PCBM), and perovskites. The slope difference could be distinguished by the presence or absence of interactions between CTAB‐capping AuNPs and surrounding media in solid states. A solid surrounding medium (C_60_) that cannot interact with CTAB or the liquid media (e.g., water or DMSO) with high fluidity produced an easy slope in a *λ*_res_ versus *n*_med_ plot. Note that we could not find any evidence in FT‐IR analysis regarding the interaction between C_60_ and CTAB that caps AuNPs (Figure S7a, Supporting Information). In contrast, the surrounding media (PCBM, bis‐PCBM, perovskites) capable of interacting with CTAB in solid states exhibited a steep slope in a *λ*_res_ versus *n*_med_ plot. Fullerene derivatives with a carbonyl functional group (e.g., bis‐PCBM and PCBM) displayed carbonyl stretching peaks shifted to lower wavenumbers in FT‐IR analysis, showing the strong interaction of the carbonyl group with the ammonium moiety of CTAB (Figure S7b–e, Supporting Information). These results imply that the dependence of *λ*_res_ on *n*_med_ can be strengthened by strong interactions between nanoparticles and surrounding media. The tightly coupled interactions in solid states may induce a stronger influence on the dielectric function of AuNPs by surrounding media in an integrated system, although a lower *n*_med_ is present in a single phase. In contrast, a loosely coupled interaction or liquid media with high fluidity may have weak or less aligned effects on the dielectric function of AuNPs.

We also investigated the chiroptical properties by checking the Mueller matrix elements related to CD (M_14_/M_41_) recorded in reflection and transmission via Mueller matrix spectroscopic ellipsometry (MMSE), as described in a previous report.^[^ ^45^ ^]^ In MMSE for ITO/AuNPs film, the circular polarization property for M_14_/M_41_ was only observed in transmission mode, which can be elucidated by intrinsic magneto‐electric coupling‐induced natural optical activity that is not associated with reflection losses (i.e., differential reflection of LCPL and RCPL) (Figure S8, Supporting Information). Therefore, the chirality of AuNPs arises from the intrinsically differentiated interaction of magnetic and electric dipoles depending on LCPL or RCPL. In MMSE for the ITO/Cs_0.05_FA_0.5_MA_0.45_Pb_0.5_Sn_0.5_I_3_ film, no circular polarization property for M_14_/M_41_ was observed as expected (Figure S9, Supporting Information). For the ITO/AuNPs/ Cs_0.05_FA_0.5_MA_0.45_Pb_0.5_Sn_0.5_I_3_ film, the circular polarization property for M_14_/M_41_ in transmission was evident with spectral shifting toward the longer wavelength compared with ITO/AuNPs (Figure S10, Supporting Information). These results imply that the chirality in an integrated system consisting of AuNPs and perovskite may arise from the intrinsic natural optical activity, similar to AuNPs; spectral resonance can be modulated by the surrounding perovskite. This suggests that such a system may be used as a CPL detector if the resonance spectrum of AuNPs modulated by the surrounding medium corresponds to the absorption range of the surrounding medium.

### 2.3 Device Performance of CPL Detectors

**Figure 3:**
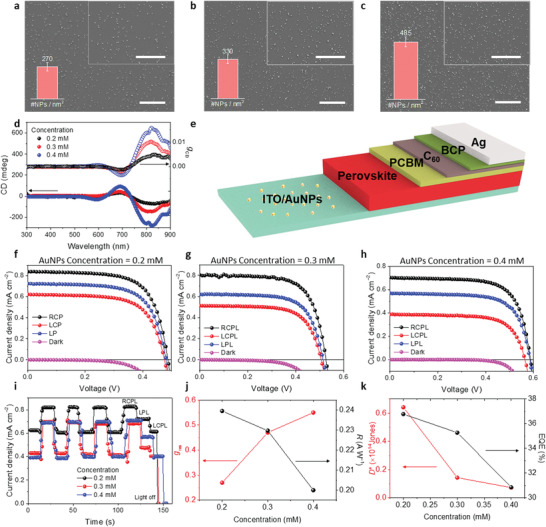
Device performance with various precursor concentrations of gold. Surface SEM micrographs of AuNPs deposited on ITO with precursor concentrations of a) 0.2, b) 0.3, and c) 0.4 mm. Inset shows higher magnitude SEM images. Scale bars in all SEM images indicate 10 µm. Nanoparticle densities expressed as the number of NPs per unit area (#NPs nm^−2^) are shown in the inset bar charts. d) CD spectra for ITO/AuNP/perovskite films with various gold precursor concentrations. e) Schematic device configuration of the CPL detector using chiral plasmonic AuNPs. *J*–*V* characteristics of CPL detectors with various gold precursor concentrations of f) 0.2, g) 0.3, and h) 0.4 mm under CPL illumination (*λ*: 808 nm, intensity: 3.5 mW cm^−2^). i) *J*–*t* curves of CPL detectors with various gold precursor concentrations. Gold precursor concentration‐dependent j) *g*_res_ (left axis) and *R* (right axis), k) *D* ^*^ (left axis) and EQE (right axis) of CPL detectors.

To elucidate the effects of chiroptical response in chiral plasmonic AuNPs‐embedded perovskite on device performance in the NIR region, we first fabricated ITO/AuNPs substrates with various initial concentrations of gold precursor. The higher precursor concentration was verified by a higher density of AuNPs on ITO, as shown in **Figure** **3**a–c. Corresponding CD spectra showed that a higher density of AuNPs on ITO induced greater degrees of CD intensities (Figure 3d). Then, we fabricated the CPL detector with the device configurations of ITO/AuNPs/Cs_0.05_FA_0.5_MA_0.45_Pb_0.5_Sn_0.5_I_3_/PCBM/C_60_/bathocuproine (BCP)/Ag (Figure 3e and Figure S11 in the Supporting Information). Recently, it was found that the mixed Pb—Sn perovskite itself produces upward band‐bending near the interface, which is behavior distinct from conventional lead‐based counterparts.^[^ ^46^ ^]^ This enables our HTL‐free configuration to maintain photovoltaic effects comparable with full‐structured solar cells. This was perfectly reflected in the current density–voltage (*J*–*V*) curve of the CPL detector, producing excellent rectifying behaviors (Figure 3f). These device characteristics enable differentiation of RCPL and LCPL under zero bias, which implies that our CPL detector can operate without external power input (i.e., self‐powered mode). To highlight this strength compared with previous reports, we performed all device measurements on CPL detectors without the application of external voltages. As shown in *J*–*V* and corresponding current density–time (*J*–*t*) curves under NIR irradiation (808 nm at 3.5 mW cm^−2^) at zero bias (Figure 3f–i), all CPL detectors showed higher photocurrents under the RCPL than under the LCPL, regardless of precursor concentrations. This result is consistent with the CD spectra of chiral AuNPs‐embedded perovskites and the CD spectroscopy‐based measurement of the difference between absorbance (*A*) of LCPL and RCPL (*A*_LCPL_–*A*_RCPL_). Non‐CPL linearly polarized light (LPL) produced values of photocurrents that were approximately intermediate between the photocurrents under RCPL and LCPL. The discrimination ability for CPL light of CPL detectors was characterized by *g*_res_ in analogy to *g*_CD_, as follows,

(3) $$g_{\mathrm{res}}=\;-\frac{2\left(R_{L}-R_{R}\right)}{\left(R_{L}+R_{R}\right)}\;$$

where *R*_L_ and *R*_R_ are the responsivities under LCPL and RCPL illumination, respectively.

The CPL detector prepared by 0.4 mm initial concentration of gold precursor showed a prominent discrimination ability for CPL light, achieving *g*_res_ of 0.55 under 808 nm CPL illumination, which is the highest *g*_res_ thus far, compared with the *g*_res_ values of chiral 2D perovskite‐based CPL detectors.^[^ ^17^, ^18^, ^19^, ^20^, ^21^, ^23^ ^]^ These results are also of great importance when considering that previous low‐dimensional perovskite‐based CPL detectors required external voltages and could not effectively detect CPL in the NIR region, thus preventing advanced applications based on NIR light absorption. Photoresponsivity (*R*), detectivity (D^*^), and external quantum efficiency (EQE) were also extracted to further characterize sensitivity to incident light in our photodetectors. Using an AuNP‐dispersed aqueous solution prepared with 0.4 mm initial gold precursor concentration, we obtained an *R* of 0.24 A W^−1^, *D* ^*^ of 7.26 × 10^12^ Jones, and EQE of 30.9% under zero bias, comparable with or slightly higher than previous chiral 2D perovskite‐based CPL detectors; these findings were highly promising in that previous results were obtained under a high external voltage. We further optimized the important characteristics (e.g., *R*, *D* ^*^, and EQE) of photodetectors by controlling the thickness of the perovskite layer, as discussed later. The higher initial concentration of gold precursor yielded the higher *g*_res_ of CPL detectors, which is consistent with the higher CD signals. In contrast, the lower *R*, *D* ^*^, and EQE were obtained with the higher concentration were obtained (Figure 3j,k), which can be attributed to the increased leakage path by the highly conductive AuNPs throughout the device; this resulted in higher dark currents (Figure S12, Supporting Information). This suggests a trade‐off between those factors and *g*_res_ because the figure of merits in a typical photodetector are closely related to the dark current. For higher concentrations of gold precursor (e.g., 0.6 or 0.8 mm), we could not obtain higher device performance in terms of *g*_res_, *R*, *D* ^*^, and EQE, compared with the values obtained from the optimized precursor concentration of gold (0.4 mm) (Figure S13, Supporting Information). At concentrations above 0.4 mm, homogenous formation of AuNPs on ITO did not occur, leading to the highly aggregated morphology of AuNPs on ITO (Figure S14, Supporting Information). This aggregation would lead to many predominant shunt paths through conductive AuNPs, degrading the fill factor (FF) of the device. The light intensity‐dependent device characteristics were also investigated as shown in Figure S15 in the Supporting Information; the corresponding *g*_res_, *R*, *D* ^*^, and EQE are summarized in Figure S16 in the Supporting Information. Under all light intensities, the detector showed a discrimination ability for CPL light under zero bias, but it showed a continuously decreasing *g*_res_ at lower light intensity. This is possibly because of the diminishing influence of the chiral plasmonic effect at the lower light intensity, which leads to the weaker amplified field by chiral plasmonic AuNPs. On the other hand, *R*, *D* ^*^, and EQE continuously increased with the lower light intensity, reaching *R* of 0.40 A W^−1^, *D* ^*^ of 1.45 × 10^13^ Jones, and EQE of 61.5% at 5 µW cm^−2^ under RCPL illumination, which is a commonly observed phenomenon in typical photodetectors.^[^ ^47^ ^]^ Additionally, the chiroptical response in visible red at around 680 nm, distinct from NIR region, implies that our detector can also be utilized for CPL detection under the wavelength. The discrimination capability of CPL in the visible red region was also verified (Figure S17, Supporting Information), where a higher photocurrent was produced under LCPL illumination than under RCPL illumination; this is consistent with the CD spectrum signal opposite to the NIR region. The lower *g*_res_ of 0.04 was obtained under 650 nm (5 mW cm^−2^) CPL illumination at zero bias because of the lower chiroptical response, compared with the NIR region.

In addition to CPL detectors using chiral AuNP‐embedded Cs_0.05_FA_0.5_MA_0.45_Pb_0.5_Sn_0.5_I_3_, we also attempted to fabricate CPL detectors with MAPbI_3_ as an active layer because the chiroptical response in the NIR region remains prominent in the chiral AuNP‐embedded MAPbI_3_ system. Despite the insignificant photocurrents of MAPbI_3_ under the excitation at 808 nm and the poor rectifying behaviors induced by downward band bending between ITO and MAPbI_3_,^[^ ^48^ ^]^ the distinction between RCPL or LCPL was feasible, yielding *g*_res_ of 0.2 and 0.01 under 808 nm (3.5 mW cm^−2^) and 650 nm (5 mW cm^−2^) CPL illumination, respectively (Figure S18, Supporting Information). These findings imply that direct detection of CPL can be achieved with various types of active layers if there is at least a small amount of absorption by the active layer in the plasmonic resonance region.

**Figure 4:**
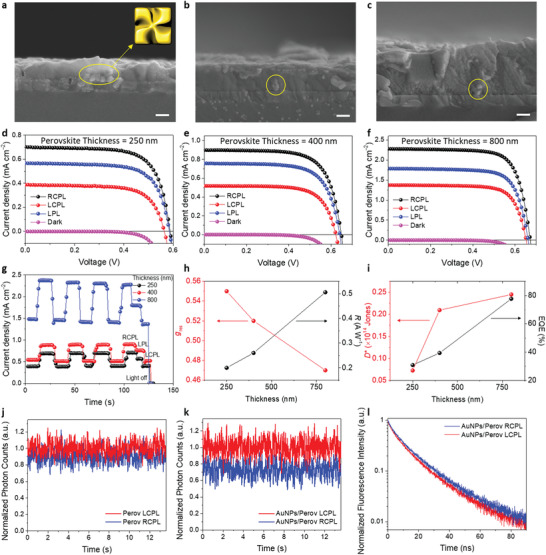
Device performance with various thicknesses of Cs_0.05_FA_0.5_MA_0.45_Pb_0.5_Sn_0.5_I_3_. SEM micrographs of a cross‐section of ITO/AuNP/perovskite with thicknesses of a) 250, b) 400, and c) 800 nm. Scale bar indicates 200 nm. *J*–*V* characteristics of CPL detectors with various perovskite thicknesses (d): 250 nm, e): 400 nm, and f) 800 nm) under CPL illumination (*λ*: 808 nm, intensity: 3.5 mW cm^−2^). g) *J*–*t* curves of CPL detectors with various perovskite thicknesses. Perovskite thickness‐dependent h) *g*_res_ (left axis) and *R* (right axis), i) *D* ^*^ (left axis) and EQE (right axis) of CPL detectors. Normalized fluorescent photon counts of MAPbI_3_ films j) without or k) with AuNPs under CPL excitation (*λ*: 633 nm). l) Normalized decay profile for the fluorescent intensity of MAPbI_3_ film with AuNPs under CPL excitation.

To further characterize the device performance of CPL detectors, we investigated thickness‐dependent device performance with a fixed precursor concentration of gold (0.4 mm). **Figure** **4**a–c shows cross‐sectional SEM images of ITO/AuNPs/ Cs_0.05_FA_0.5_MA_0.45_Pb_0.5_Sn_0.5_I_3_ with various thicknesses (250, 400, and 800 nm) of Cs_0.05_FA_0.5_MA_0.45_Pb_0.5_Sn_0.5_I_3_. AuNPs were confirmed to be embedded in an active layer where the perovskite compactly encased AuNPs without voids (yellow circles in Figure 4a–c). SEM images of the perovskite surface at various thicknesses deposited on ITO/AuNPs showed compact coverage without pinholes (Figure S19, Supporting Information). Figure 4d–f shows the *J*–*V* curves of CPL detectors under 808 nm CPL illumination at 3.5 mm cm^−2^ light intensity. Compared with the CPL detector containing 250 nm‐thick perovskite, the thicker perovskites produced larger photocurrents. Figure 4g shows the verification of CPL detection at zero bias producing a higher photocurrent under RCPL illumination than under LCPL illumination. A slightly decreasing *g*_res_ was obtained with thicker perovskites, possibly because of the reduced contribution of chiral plasmonic enhancement by AuNPs to the thicker active layer. However, as a photodetector, improved performance was achieved with thicker active layers (Figure 4h,i). For the CPL detector using 800 nm‐thick perovskite, the highest *R* of 0.51 A W^−1^, *D* ^*^ of 2.45 × 10^13^ Jones, and EQE of 77.7% were obtained under zero bias. The improved photo‐sensitivity probably originated from the higher photocurrents and the reduced leakage currents because of thicker active layers. Especially, *D* ^*^ was much higher compared with the state‐of‐the‐art chiral 2D perovskite‐based CPL detectors and even comparable with 3D MAPbI_3_‐based CPL‐insensitive photodetectors (≈10^12^–10^14^ Jones).^[^ ^49^, ^50^ ^]^ These findings could be facilitated by chiral plasmonic AuNP‐assisted CPL detection solely through the use of 3D perovskites with superior optoelectronic properties compared with 2D counterparts. Although *g*_res_ decreased slightly from 0.55 at a thickness of 250 nm to 0.47 at a thickness of 800 nm, it remained the highest value reported thus far, compared with chiral 2D perovskites.

We investigated the effects of chiral plasmonic AuNPs on the charge carrier dynamics of perovskite films by performing steady‐state and time‐resolved photoluminescence (TRPL) analysis of the perovskite (MAPbI_3_) films with or without chiral AuNPs, as shown in Figure 4j–l. MAPbI_3_ was utilized instead of Cs_0.05_FA_0.5_MA_0.45_Pb_0.5_Sn_0.5_I_3_ because of the NIR wavelength detection limit in our PL system. For the perovskite film without chiral AuNPs, a negligible difference in radiative photon counts was observed under 633 nm RCPL or LCPL excitation (Figure 4j). A noticeable distinction in radiative photon counts was observed depending on the CPL direction for the chiral AuNP‐embedded perovskite film (Figure 4k). Considering that selective near‐field electric field enhancement is induced for specific polarization direction in chiral plasmonic AuNPs upon surface plasmon excitation, the perovskite directly in contact with AuNPs would comply with such selective electric field enhancement. Consequently, distinct degrees of fluorescence intensity can be induced in perovskites for parts of the CPL direction. Stronger fluorescence under LCPL illumination than under RCPL illumination may illustrate a larger exciton population under 633 nm LCPL illumination, which is consistent with the CD spectrum of AuNPs/MAPbI_3_ film. On TRPL analysis, in contrast to exhibiting identical decay time profile in the perovskite without chiral AuNPs, AuNP‐embedded perovskite displayed dissimilar fluorescence decay rate according to the rotation direction of the circular polarization of incident light (Figure 4l and Figure S20 in the Supporting Information). An increase in fluorescence intensity and a decrease of lifetime might be responsible for metal‐enhanced fluorescence, including an increase in the excitation rate because of local field enhancement and fluorophore‐metal interaction.^[^ ^51^, ^52^ ^]^

### 2.4 Application to Flexible CPL Detectors

**Figure 5:**
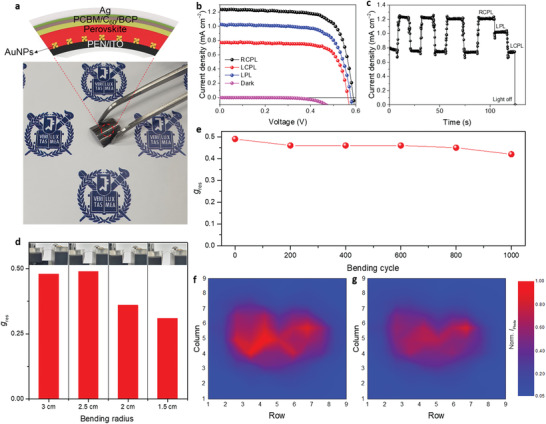
Device performance of the flexible CPL detector array. a) Photograph and schematic device configuration of the flexible CPL detector array. b) *J*–*V* and c) corresponding *J*–*t* curve for the flexible device under CPL illumination. d) *g*_res_ of the flexible device with various bending radii. *g*_res_ was obtained after repetitive bending for 100 cycles with each bending radius. Inset photographs show the bending degree for each bending radius. e) *g*_res_ of the flexible device in bending test for 1000 cycles with the fixed bending radius of 2.5 cm. Photocurrent mapping of the photosensor matrix exposed to a heart‐shaped laser source (*λ*: 808 nm, intensity: 3.5 mW cm^−2^) under f) RCPL illumination or g) LCPL illumination.

A flexible CPL 9 × 9 array detector was constructed on a PEN/ITO substrate as shown in **Figure** **5**a. The excellent rectifying behavior and the discrimination ability for CPL were completely maintained, achieving a *g*_res_ of 0.49 under 808 nm CPL illumination (3.5 mW cm^−2^) at zero bias (Figure 5b,c). In addition, a comparable *R* of 0.35 A W^−1^, *D* ^*^ of 1.54 × 10^13^ Jones, and EQE of 54.2% were obtained. The mechanical flexibility of the device was examined by conducting bending tests at various bending radii, where the bending was performed for 100 cycles at each radius (Figure 5d). The flexible device showed excellent reproducibility up to a bending radius of 2.5 cm, without degrading *g*_res_, while slight decreases in *g*_res_ were observed for a bending radius over 2.5 cm (Figure S21, Supporting Information). The prominent flexibility and durability were verified with a repetitive bending test for 1000 cycles at the critical 2.5 cm bending radius (Figure 5e and Figure S22 in the Supporting Information). The flexible device retained almost 90% of its initial *g*_res_ after the 1000 cycle test, which suggests that the flexible CPL array possesses excellent mechanical durability and can be effectively utilized for flexible device applications. The photomapping characteristic of the fabricated flexible CPL 9 × 9 array detector is shown in Figure 5f,g. The center of the sensing matrix was directly exposed to a heart‐shaped laser source of 808 nm CPL illumination. Consequently, the light‐exposed region was expressed using a 2D histogram, as a function of the produced photocurrent. The higher photocurrent, produced under RCPL illumination than under LCPL illumination, was revealed as a deeper red color in the photomapping. These results represent a valuable step toward integrated 3D perovskite‐based CPL detector applications capable of real‐time detection of NIR CPL.

## 3 Conclusion

In summary, we have devised a hybrid system composed of chiral plasmonic AuNPs and mixed Pb—Sn perovskite to directly detect CPL, especially in the NIR wavelength region. We found that the chiral optical response manifested by chiral plasmonic nanoparticles, in conjunction with intact 3D perovskites, outperforms typical chiral 2D perovskites that use molecular chiral systems. The chiroptical response of chiral plasmonic AuNPs could be tuned by adjusting the refractive index of the surrounding medium, presumably because of dynamic modulation in the dielectric function of AuNPs depending on surrounding media. The spectral matching between AuNPs plasmonic resonance and perovskite absorption allowed effective CPL discrimination in the NIR spectral region. We adopted an effective HTL‐free device configuration for low‐power consumption devices that could fully retain photovoltaic effects, enabling a self‐powered mode CPL detection. The resulting CPL detectors based on chiral AuNP‐embedded perovskite films exhibited remarkable chirality with a high *g*_res_ of 0.55 under zero‐bias, the highest value compared with recently reported chiral 2D perovskite‐based CPL detectors. An outstanding *D* ^*^ greater than 2 × 10^13^ Jones was also obtained, the highest among values reported for perovskite‐based CPL detectors. Furthermore, we successfully implemented a flexible CPL detector array with excellent durability that can be applied to the real‐time detection of NIR CPL, the first proof‐of‐concept demonstration of NIR CPL photomapping. Compared with conventional perovskite‐based CPL detectors that use low‐dimensional perovskites with poor optoelectronic properties, our results provide a versatile design rule for efficient CPL detectors, thereby preserving the superior optoelectronic properties of 3D perovskites. Our findings will facilitate the fabrication of high‐performance self‐powered CPL detectors that can be used for next‐generation chiral optoelectronics.

## 4 Experimental Section

### Materials

Tin iodide (SnI_2_), C_60_, and BCP were purchased from Alfa Aesar. Formamidinium iodide (FAI) and methylammonium iodide (MAI) were purchased from GreatCell Solar. Cesium iodide (CsI), lead iodide (PbI_2_), lead thiocyanate (Pb(SCN)_2_), bis‐PCBM, CTAB, gold(III) chloride solution (HAuCl_4_), ascorbic acid, *L*‐glutathione, anhydrous *N*,*N*‐dimethyl formamide (DMF), DMSO, *o*‐chlorobenzene (ODCB), anhydrous toluene, and chlorobenzene (CB) were purchased from Sigma‐Aldrich. Tin fluoride (SnF_2_) was obtained from Acros Organics. PCBM was obtained from EM‐index. All chemicals were used as received without further purification.

### Synthesis of Chiral Plasmonic AuNPs

Octahedral seed nanoparticles were synthesized in accordance with a previously reported method.^[^ ^53^ ^]^ After the washing process, particles were redispersed in CTAB solution. For the 432 helicoid III synthesis, a growth solution was prepared by adding 0.8 mL of 100 mm CTAB, and 10 mm HAuCl_4_ into 3.95 mL deionized water. 0.475 mL of 100 mm ascorbic acid solution was injected for Au^3+^ reduction. The growth of chiral nanoparticles was initiated by injecting 5 µL of a 5 mm L‐glutathione solution and adding 50 µL octahedral gold seed nanoparticles. The growth solution was placed in a 30 °C bath for 2 h, and the solution gradually turned blue with extensive light scattering. The solution was centrifuged twice and redispersed in 1 mm CTAB solution for further use.

### Perovskite Precursor Solution Preparation

Thin films (perovskite thickness of approximately 250 nm) were prepared with a mixed Pb—Sn perovskite precursor solution with the chemical formula Cs_0.05_FA_0.5_MA_0.45_Pb_0.5_Sn_0.5_I_3_ by dissolving 153.7 mg of SnI_2_, 70.9 mg of FAI, 6.5 mg of SnF_2_, 190.2 mg of PbI_2_, 59 mg of MAI, 10.7 mg of CsI, and 4 mg of Pb(SCN)_2_ into DMF:DMSO (800 µL:200 µL). Thick perovskite films of approximately 400 or approximately 800 nm were prepared using the same procedure, except the amount of each ingredient was increased by 1.5 or 2.5‐fold, respectively. The precursor solution for fabricating MAPbI_3_‐based devices was obtained by preparing a 0.825 mm solution composed of PbI_2_ and MAI at a molar ratio of 1.05:1 in DMF:DMSO (9:1 volume ratio).

### Device Fabrication

ITO‐coated glasses were rinsed sequentially with deionized water, acetone, and isopropyl alcohol. Rinsed substrates were treated with O_2_ plasma for 1 min at 100 W. The chiral plasmonic AuNPs, dispersed in an aqueous solution, were washed several times using centrifugation at 12 000 rpm for 9 min, followed by resuspension. Drop‐casting of AuNPs onto an ITO substrate was conducted by dropping AuNP‐dispersed aqueous solution on the ITO held at 36 °C on a hot plate. A petri dish was placed upside down on the substrate to mitigate the rapid aggregation of AuNPs during drying. On the ITO/AuNP substrate, the perovskite precursor solution was spin‐coated under two‐step coating conditions: 1000 rpm for 10 s and 3500 rpm for 40 s, followed by annealing at 100 °C for 5 min. Toluene was used as the antisolvent for the second spin‐coating process. The PCBM solution (5 mg mL^−1^) in ODCB was filtered and spin‐coated onto the perovskite film at 2000 rpm for 60 s. Then, 20 nm‐thick C_60_, 7 nm‐thick bathocuproine, and 100 nm‐thick Ag layers were thermally evaporated under vacuum (< 10^−6^ mbar) in sequence through a shadow mask. The active area of the robust device was 0.015 cm^2^. Flexible CPL detector arrays were fabricated using the same procedures, except PEN/ITO substrate was utilized instead of robust ITO substrate; a small active area of 0.04 mm^2^ was maintained. For devices based on MAPbI_3_, a MAPbI_3_ precursor solution was filtered through a 0.2‐µm hydrophobic filter and spin‐coated at 4000 rpm for 50 s, then dried at 100 °C for 10 min. Anhydrous CB was dropped onto the rotating substrate 8 s after the initiation of spin coating for antisolvent treatment. Transport layers and electrodes were deposited using the same procedures as for mixed Pb—Sn perovskite‐based devices.

### Film and Device Characterization

X‐ray diffraction analyses were conducted using an X‐ray diffractometer (Smart Lab, Rigaku) with Cu K*α* radiation (*λ* = 1.5406 Å) at 40 kV/40 mA. SEM micrographs were obtained using a field emission scanning electron microscope (JSM‐7800F Prime, JEOL). Absorption spectra were measured using an ultraviolet/visible light spectrophotometer (V‐770, JASCO). FT‐IR was measured using an FT‐IR spectrometer (TENSOR27, Bruker). The refractive index and Mueller matrix were extracted using spectroscopic ellipsometry (RC2, J.A. Woollam). *J*–*V* and *J*–*t* curves were measured under vacuum using a Keithley 4200‐SCS semiconductor parametric analyzer. CPL illumination was generated through a linear polarizer and a quarter‐wave plate (Thorlabs) that were installed between the light source and samples. Light intensity‐dependent device characteristics were measured by modulating neutral density filters with various optical densities. Steady‐state and TRPL analyses were carried out using a time‐correlated single‐photon‐counting board (SPC‐150, Becker & Hickl), a 633 nm picosecond‐pulsed diode laser (Picoquant), and an APD (MPD) detector with a suitable bandpass filter. The CPL direction of the laser light was controlled by wave plate rotation. CD spectroscopy was measured using a CD spectropolarimeter (J‐815, JASCO). The anisotropy factor (*g*_CD_), indicating the ratio of CD to conventional absorption, was calculated from the following equation,

(4) $$g_{\mathrm{CD}}=\;2\;\frac{A_{L}-A_{R}}{A_{L}+A_{R}}=\frac{\mathrm{CD}}{32980\times A}\;$$

where *A*_L_ is the absorption for LCPL, *A*_R_ is the absorption for RCPL, CD is the extracted value from CD spectroscopy, and *A* is the absorbance of the sample.

### Calculation of Important Characteristics of Photodetectors

The photo‐sensitivity of the device, *R* was calculated using the following equation,

(5) $$R\;=\frac{I_{\mathrm{light}}-I_{\mathrm{dark}}}{P_{\mathrm{inc}}}\;$$

where *I*_light_ is the current produced under illumination, *I*_dark_ is the dark current, and *P*_inc_ is the incident light intensity. EQE was defined as the ratio of the number of photogenerated carriers to the number of incident photons in the following equation,

(6) $$\mathrm{EQE}\;=\frac{\left(I_{\mathrm{light}}-I_{\mathrm{dark}}\right)hc}{eP_{\mathrm{int}}A\lambda }$$

where *P*_int_ is the incident power density, *e* is the elementary charge, *A* is the effective area, *h* is the Planck's constant, *c* is the speed of light, and *λ* is the wavelength. Detectivity usually describes the smallest detectable signal, as follows,

(7) $$D^{*}=\frac{\sqrt{A}}{\mathrm{NEP}}$$

(8) $$\mathrm{NEP}\;=\frac{\sqrt{{\bar{I}}_{n}^{2}}}{R\sqrt{\Delta f}}\;$$

where NEP is the noise equivalent power, ${\bar{I}}_{n}^{2}$ is the measured noise current, and Δ*f* is the bandwidth. If the major limit to detectivity is shot noise from the drain current under dark conditions, *D* ^*^ can be simplified as follows,

(9) $$D^{*}=\frac{R}{\sqrt{2e\cdot I_{\mathrm{dark}}/A}}\;$$

## Conflict of Interest

The authors declare no conflict of interest.

## Supporting information

Supporting Information

## Data Availability

The data that support the findings of this study are available in the supplementary material of this article.
