# Supplementary material for: Ultrasensitive Near‐Infrared Circularly Polarized Light Detection Using 3D Perovskite Embedded with Chiral Plasmonic Nanoparticles
Source: Adv Sci (Weinh). 2022 Jan 2;9(5):2104598. doi: 10.1002/advs.202104598 (PMC8844506; doi:10.1002/advs.202104598)
Supplement: Supplementary file 1 — Supporting Information [file ADVS-9-2104598-s001.pdf]

ADVANCED SCIENCE
Open Access

Supporting Information

for *Adv. Sci.,* DOI: 10.1002/advs.202104598

Ultrasensitive Near-Infrared Circularly Polarized Light Detection Using 3D Perovskite Embedded with Chiral Plasmonic Nanoparticles

Hongki Kim, Ryeong Myeong Kim, Seok Daniel Namgung, Nam Heon Cho, Jung Bae Son, Kijoon Bang, Mansoo Choi, Seong Keun Kim, Ki Tae Nam, Jong Woo Lee, and Joon Hak Oh**

Table of Contents

Different drop-casting procedures of AuNP-dispersed aqueous solution on ITO glass (Figure S1) -- S4

Surface SEM images of perovskite films deposited on ITO/AuNP substrate (Figure S2) ----- S5

Surface SEM images of perovskite films prepared by mixing perovskite precursor solution and AuNP solution (Figure S3) --- S6

CD spectra of ITO/AuNPs, ITO/AuNPs/$Pb_{0.5}Sn_{0.5}I_2$, and $CsPb_{0.5}Sn_{0.5}I_3$ films (Figure S4) --- S7

CD spectra of AuNPs with various surrounding media deposited on ITO/AuNP substrate and their absorbance (Figure S5) -- S8

n, *k* for each various surrounding media (Figure S6) --- S9

FT-IR spectroscopic analysis of C_{60}, PCBM, and bis-PCBM deposited on ITO/AuNPs substrate (Figure S7) -- S10

MMSE data recorded in reflection and transmission for ITO/AuNP film with various incident angles (Figure S8) -- S11

MMSE data recorded in reflection and transmission for ITO/$Cs_{0.05}FA_{0.5}MA_{0.45}Pb_{0.5}Sn_{0.5}I_3$ film with various incident angles (Figure S9) -- S12

MMSE data recorded in reflection and transmission for ITO/AuNP/ $Cs_{0.05}FA_{0.5}MA_{0.45}Pb_{0.5}Sn_{0.5}I_3$ film with various incident angles (Figure S10) ------------------ S13

Cross-sectional SEM image of the whole device (Figure S11) ---------------------------------- S14

Dark currents in CPL detectors with various gold precursor concentrations (Figure S12) --- S15

Device performance of CPL detectors with various gold precursor concentrations (Figure S13) -- - S16

Surface SEM images of ITO/AuNPs prepared with various gold precursor concentrations (Figure S14) --- S17

Light intensity-dependent device performance (Figure S15) ------------------------------------ S18

Light intensity-dependent device performance (Figure S16) ------------------------------------ S19

Device performance of CPL detector under 650 nm CPL illumination (Figure S17) --------- S20

Device performance of CPL detector using $MAPbI_3$ as an active layer under 808 nm or 650 nm CPL illumination (Figure S18) --- S21

Surface SEM images of perovskite deposited on ITO/AuNP substrate with various perovskite thicknesses (Figure S19) --- S22

Normalized fluorescence decay profiles for $MAPbI_3$ film without AuNPs under 633 nm CPL excitation (Figure S20) --- S23

J–*t* curves of flexible CPL detectors under 808 nm CPL illumination at various bending radii (Figure S21) -- S24

J–*t* curves of flexible CPL detectors under 808 nm CPL illumination after following repetitive bending tests with a fixed bending radius of 2.5 cm (Figure S22) ------------------------------ S25

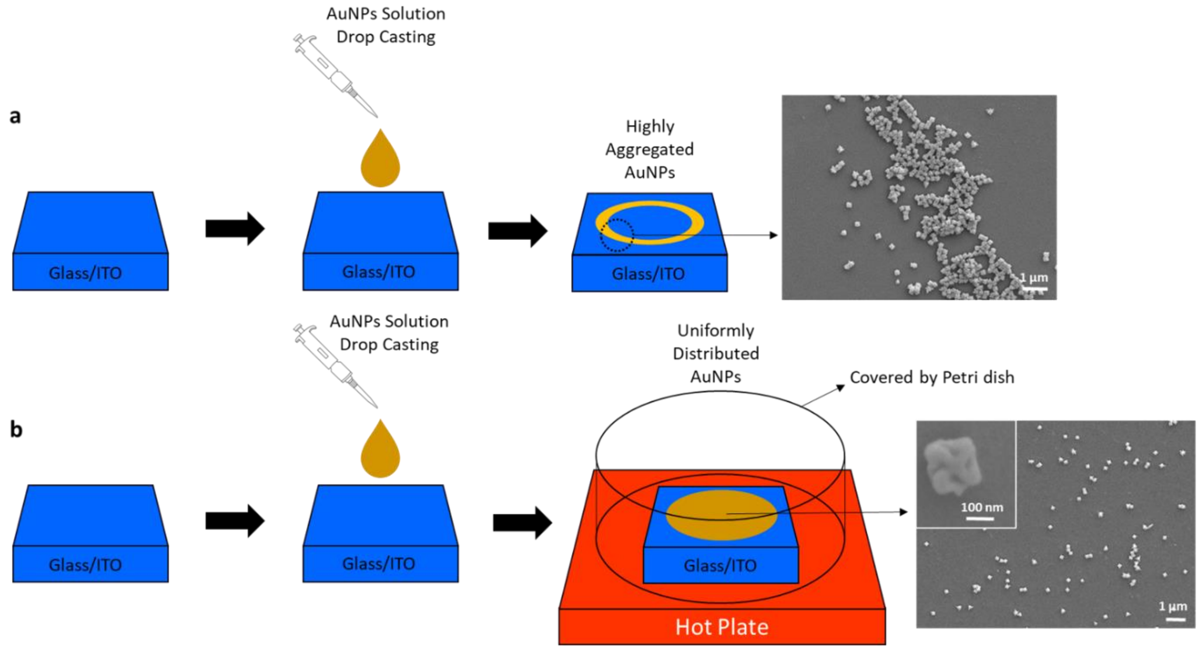

Figure S1. Different drop-casting procedures of AuNP-dispersed aqueous solution on ITO glass. (a) The typical drop-casting procedure, where the droplet of AuNP-dispersed aqueous solution was dried under ambient conditions, produced highly aggregated ring-like distribution of AuNPs on ITO substrate. (b) The modified drop-casting procedure, where the substrate was covered by an upside-down petri dish under a constant temperature while drying on a hot plate, produced a homogenous distribution of AuNPs on ITO. Each SEM image shows the morphologies resulting from the drop-casting procedures.

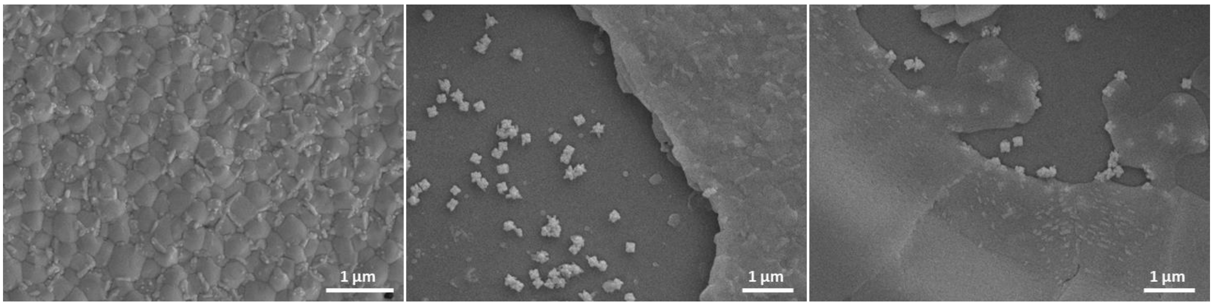

Figure S2. Surface SEM images of perovskite films deposited on ITO/AuNP substrate. The ITO/AuNP substrate is fully covered by perovskite (left) and partially covered by perovskite (middle and right).

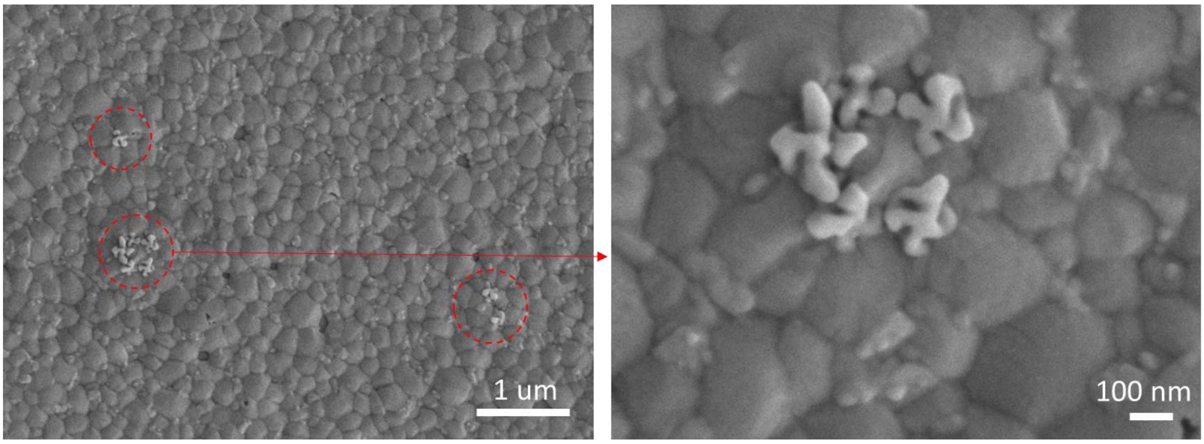

Figure S3. Surface SEM images of perovskite films prepared by mixing perovskite precursor solution and AuNP solution, where DMSO replaced the solvent in the AuNP-dispersed aqueous solution.

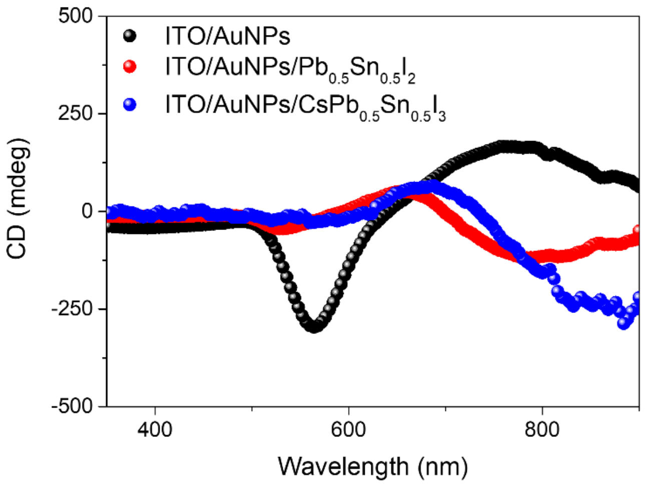

Figure S4. CD spectra of ITO/AuNPs, ITO/AuNP/$Pb_{0.5}Sn_{0.5}I_2$, and $CsPb_{0.5}Sn_{0.5}I_3$ films.

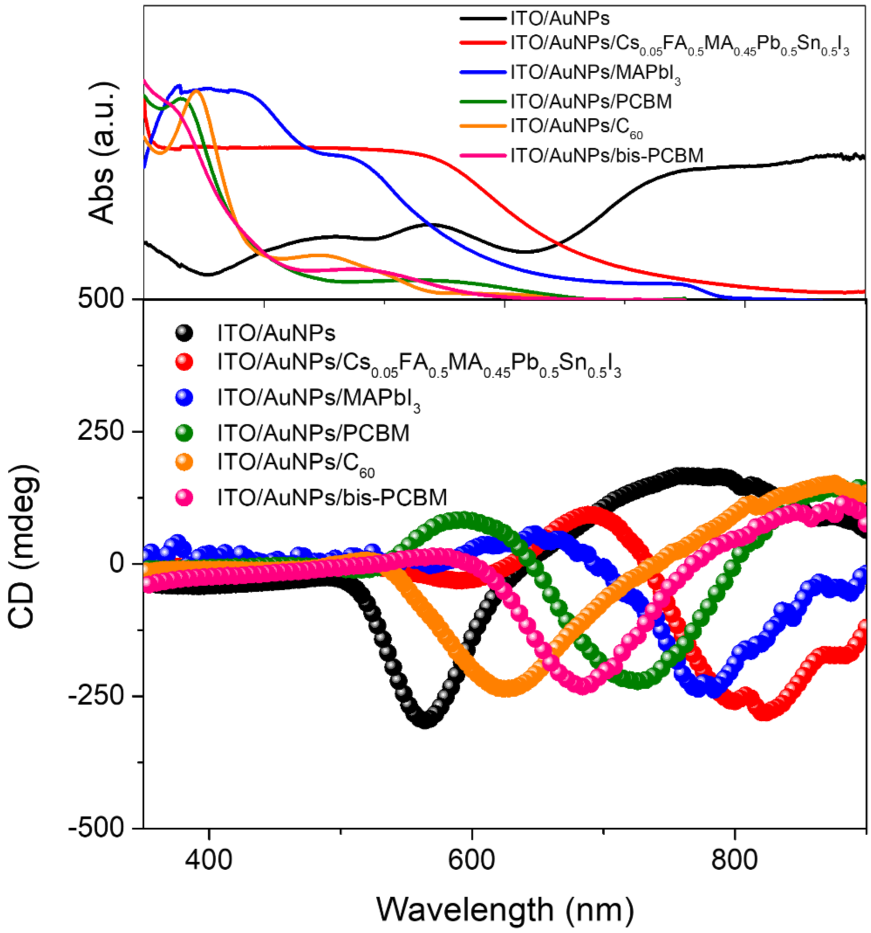

Figure S5. CD spectra (bottom) of AuNPs with various surrounding media deposited on ITO/AuNP substrate and their absorbance (top).

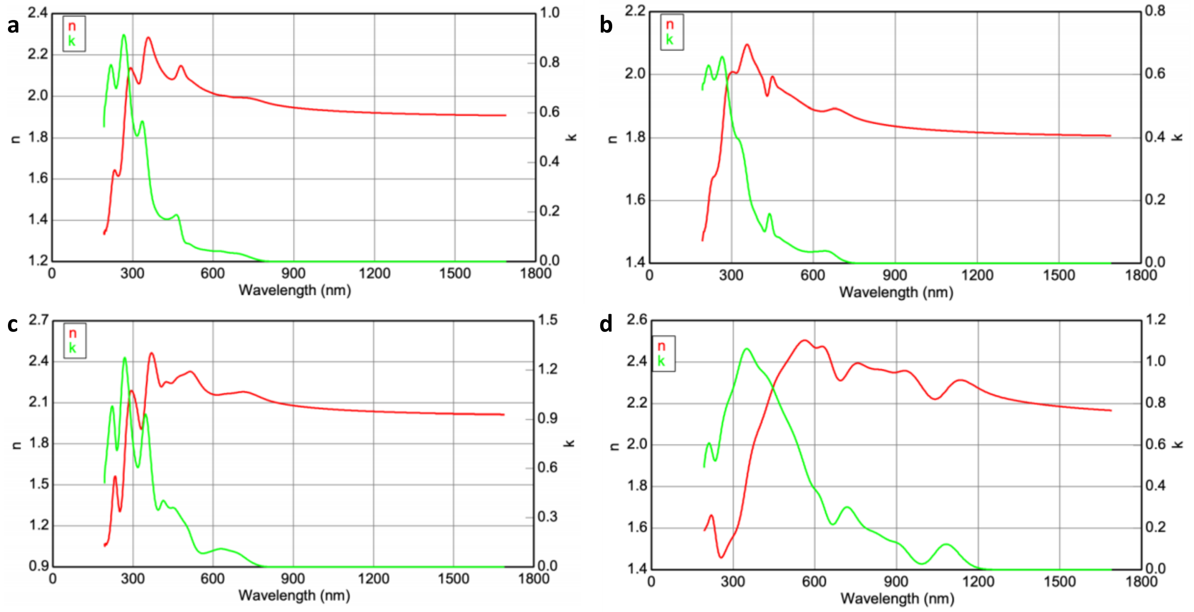

Figure S6. *n*, *k* for various surrounding media. (a) PCBM, (b) bis-PCBM, (c) C_{60}, and (d) $Cs_{0.05}FA_{0.5}MA_{0.45}Pb_{0.5}Sn_{0.5}I_3$ films on ITO.

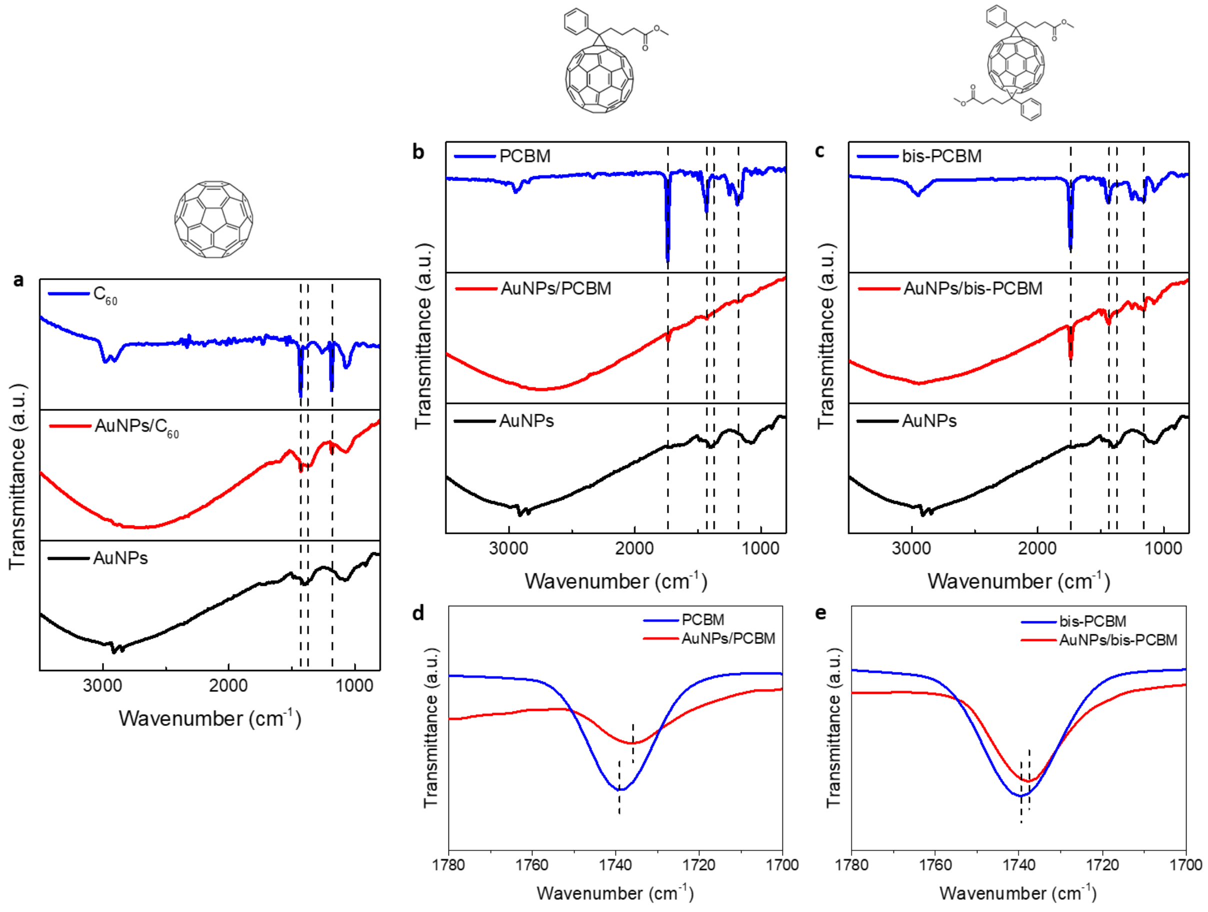

Figure S7. FT-IR spectroscopic analysis of (a) C_{60}, (b) PCBM, and (c) bis-PCBM deposited on ITO/AuNPs substrate. FT-IR spectra of regions specified for the carbonyl vibrational peak for (d) PCBM and (e) bis-PCBM.

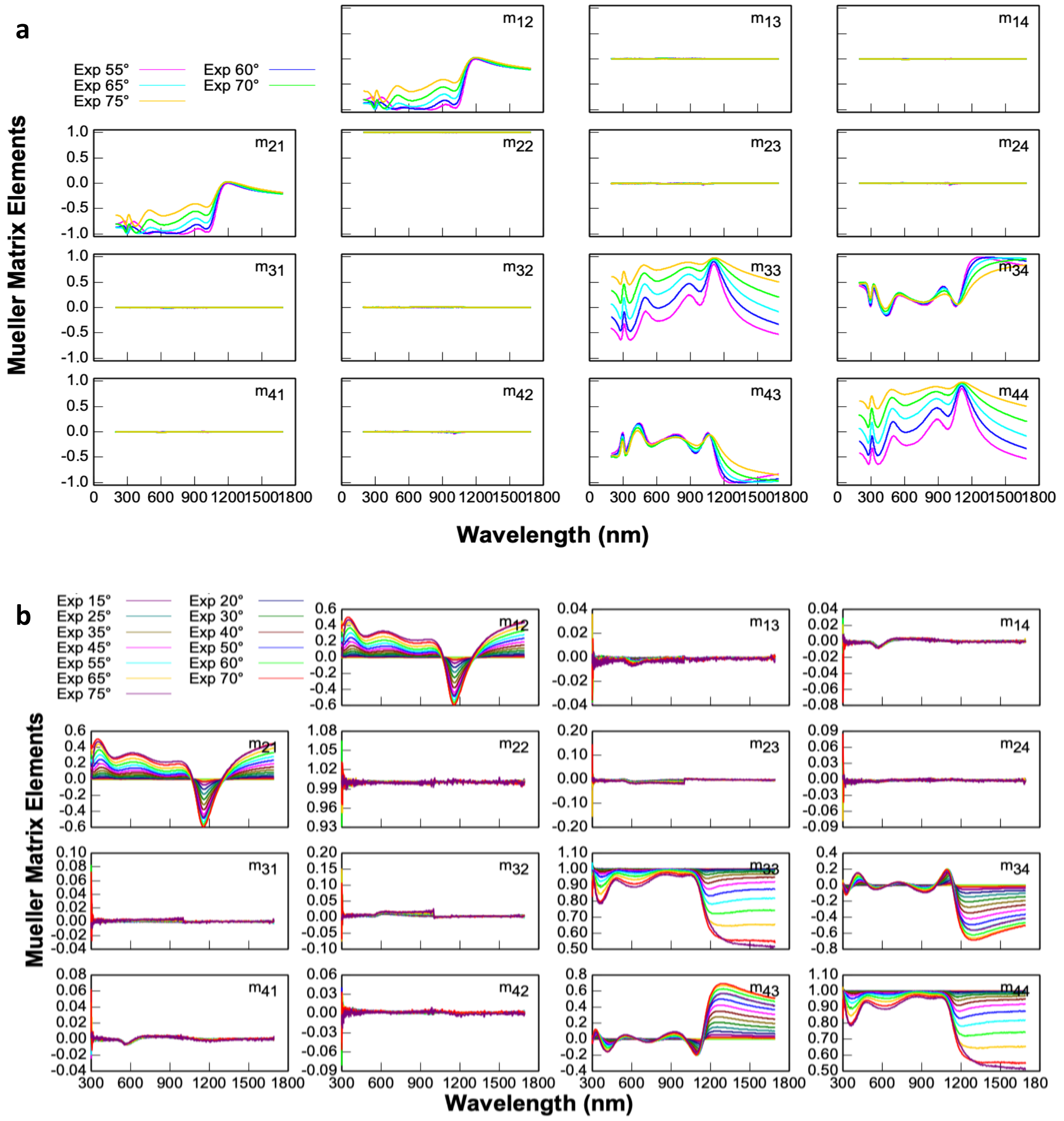

Figure S8. MMSE data recorded in (a) reflection and (b) transmission modes for ITO/AuNP film with various incident angles.

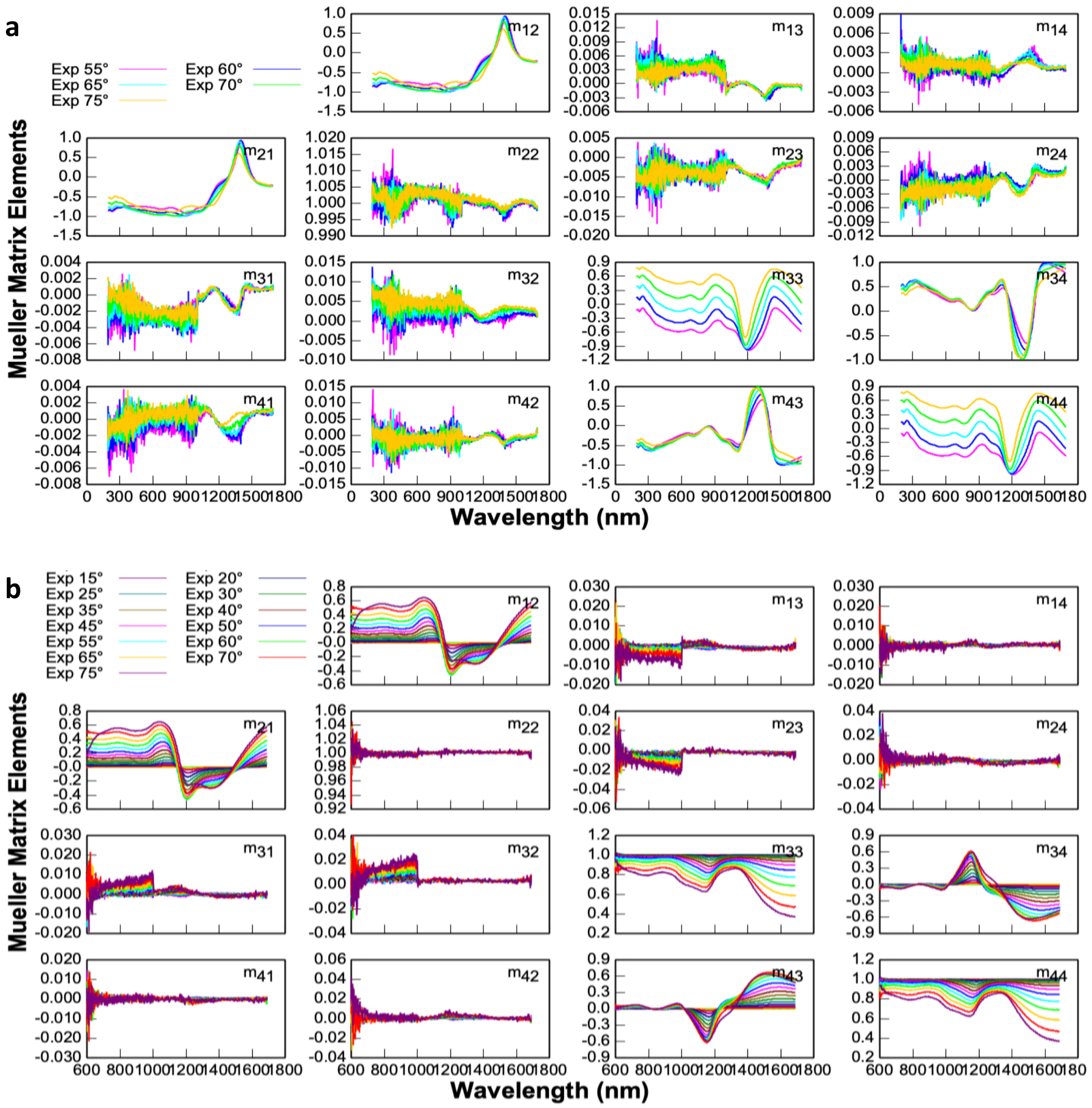

Figure S9. MMSE data recorded in (a) reflection and (b) transmission modes for ITO/$Cs_{0.05}FA_{0.5}MA_{0.45}Pb_{0.5}Sn_{0.5}I_3$ film with various incident angles.

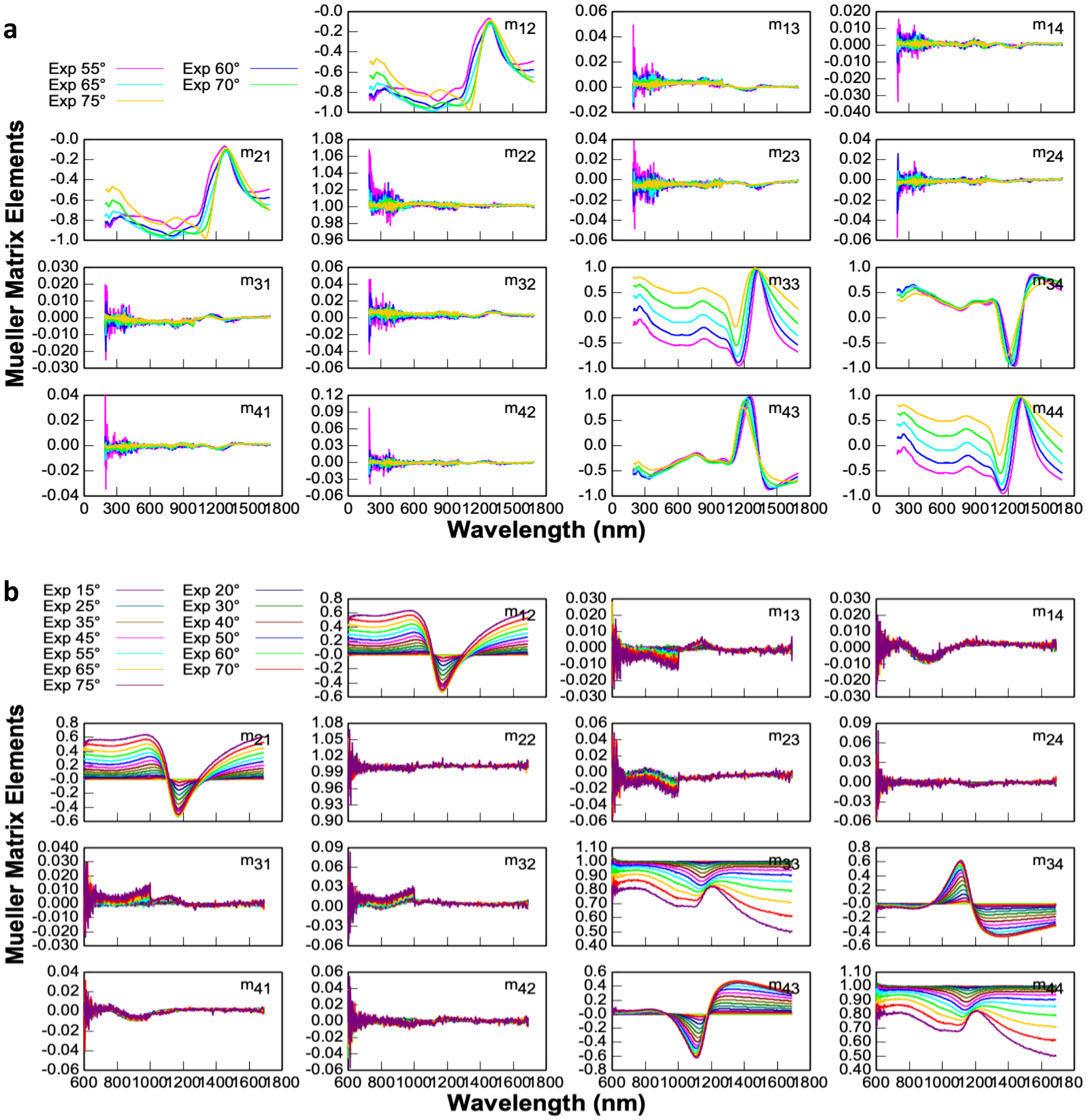

Figure S10. MMSE data recorded in (a) reflection and (b) transmission modes for ITO/AuNPs/$Cs_{0.05}FA_{0.5}MA_{0.45}Pb_{0.5}Sn_{0.5}I_3$ film with various incident angles.

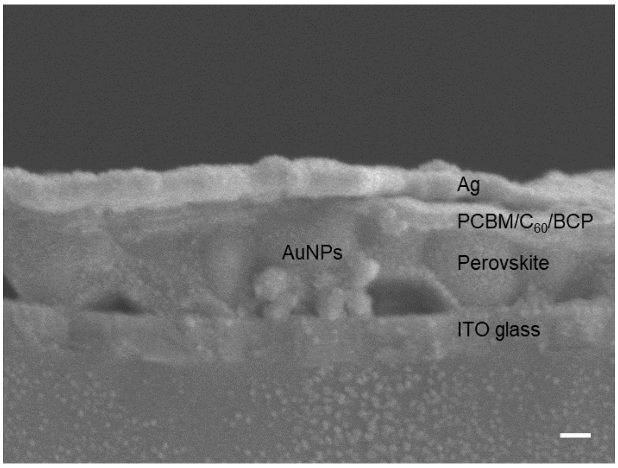

Figure S11. Cross-sectional SEM image of the whole device. Inset scale bar indicates 100 nm.

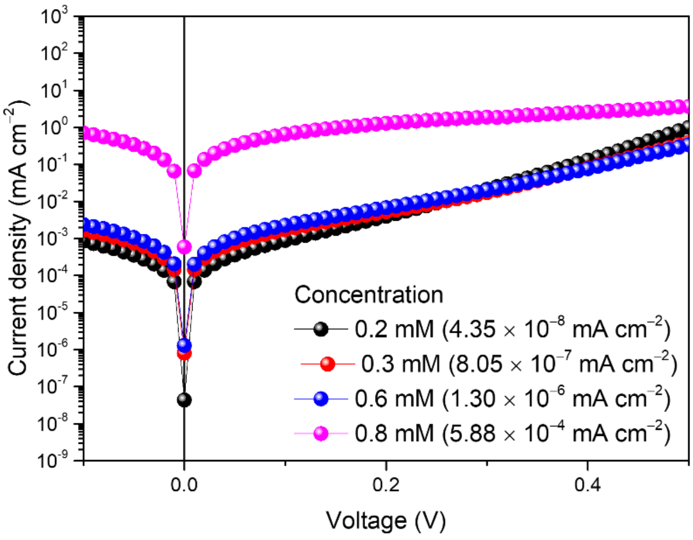

Figure S12. Dark currents in CPL detectors with various gold precursor concentrations. The exact values of dark current densities at 0 V depending on the concentrations of gold precursor were indicated in the figure.

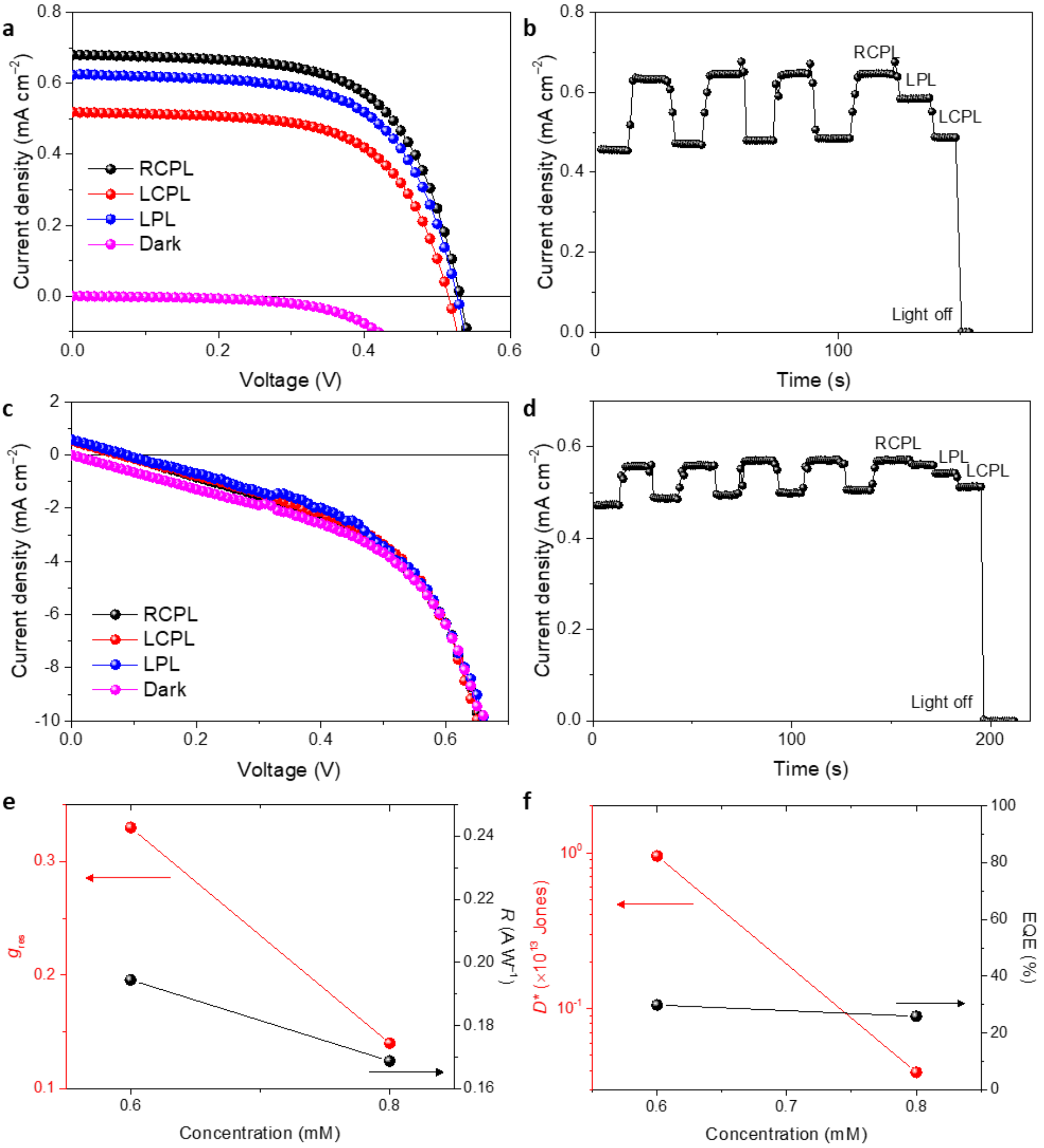

Figure S13. (a) *J–V* characteristics and (b) the corresponding *J–t* curve of CPL detectors with 0.6 mM gold precursor concentration under CPL illumination. (c) *J–V* characteristics and (d) the corresponding *J–t* curve of CPL detectors with 0.8 mM gold precursor concentration under CPL illumination. Gold precursor concentration dependent (e) g_{res} (left axis) and R (right axis), (f) D^* (left axis) and EQE (right axis) of CPL detectors.

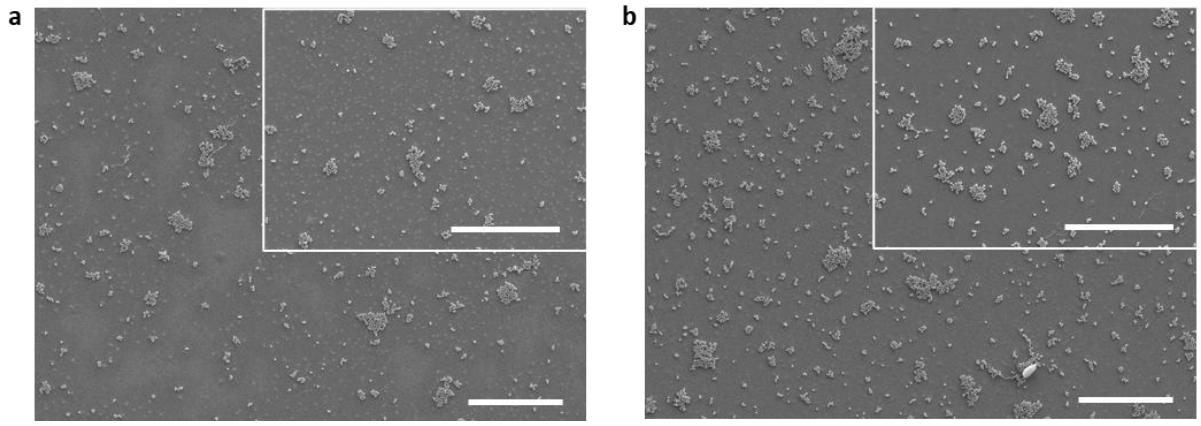

Figure S14. Surface SEM images of ITO/AuNPs prepared with different gold precursor concentrations: (a) 0.6 mM or (b) 0.8 mM. SEM images in the inset of each micrograph show the higher magnitude SEM images. Scale bars in all SEM images indicate 10 μm.

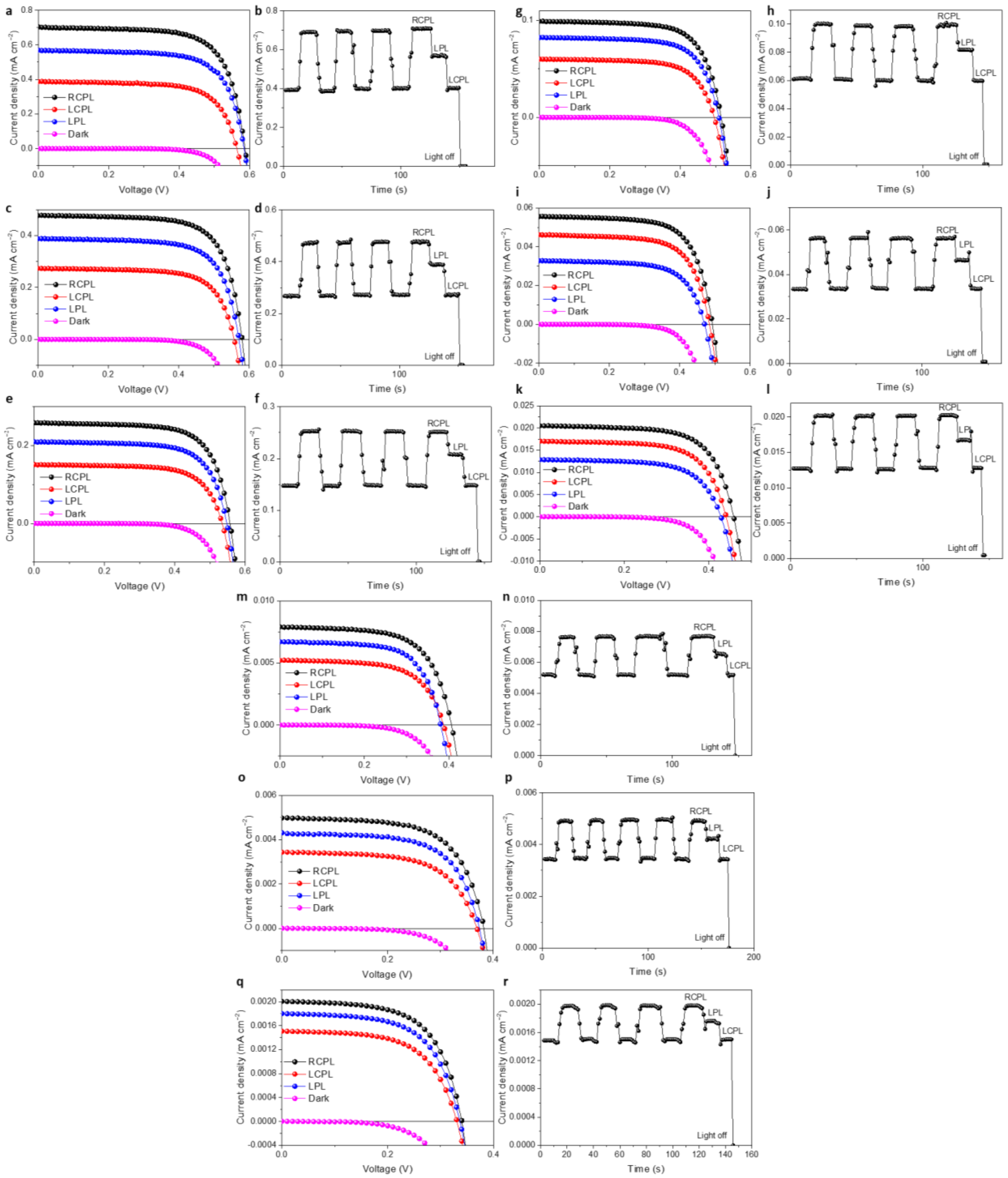

Figure S15. Light intensity dependent device performance. *J–V* characteristics and the corresponding *J–t* curve of CPL detectors with various light intensities under 808 nm CPL illumination. (a, b) 3.5 mW cm^{-2}, (c, d) 2.2 mW cm^{-2}, (e, f) 1.2 mW cm^{-2}, (g, h) 0.5 mW cm^{-2}, (i, j) 0.23 mW cm^{-2}, (k, l) 70 μW cm^{-2}, (m, n) 25 μW cm^{-2}, (o, p) 16 μW cm^{-2}, and (q, r) 5 μW cm^{-2}.

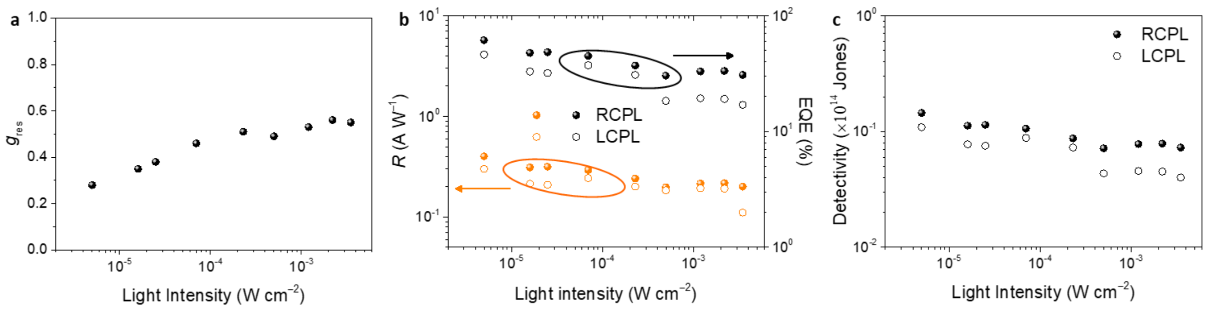

Figure S16. Light intensity dependent device performance. (a) Light intensity dependent g_{res}. (b) Light intensity dependent R (left axis) and EQE (right axis). (c) Light intensity dependent D^{*}.

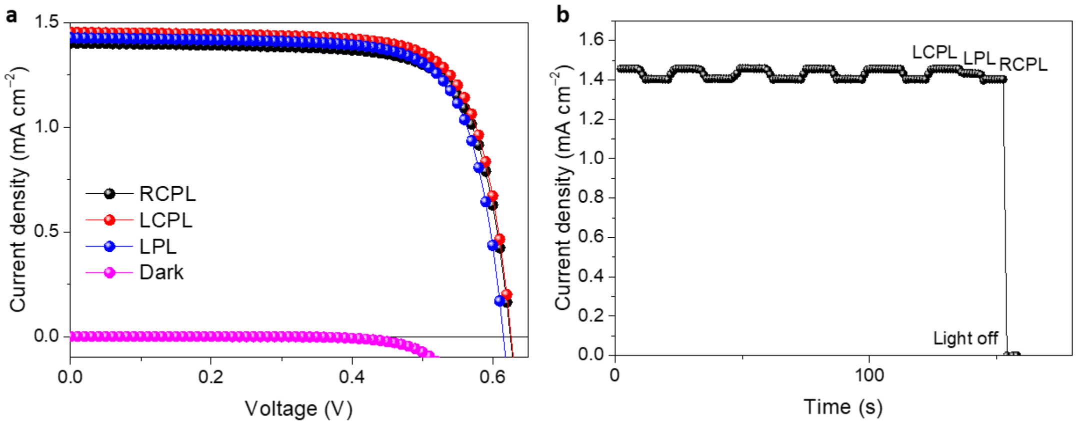

Figure S17. Device performance of CPL detector under 650 nm CPL illumination. (a) *J–V* characteristics and (b) the corresponding *J–t* curve of the CPL detector.

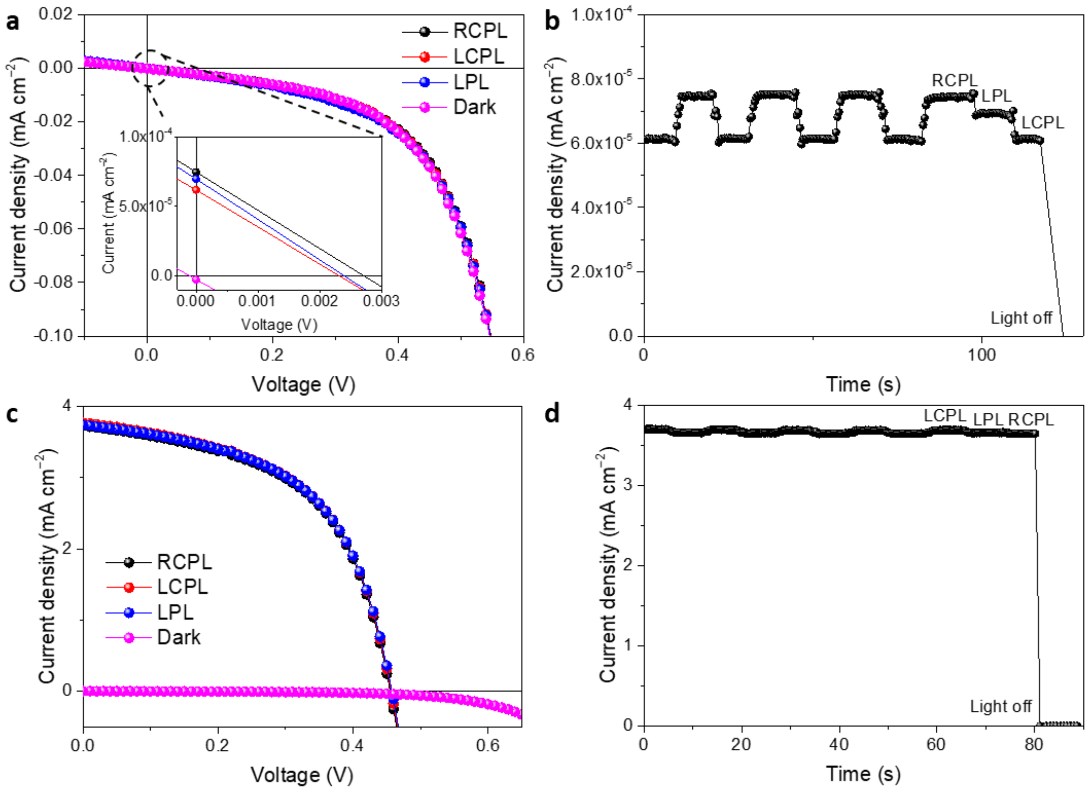

Figure S18. Device performance of CPL detector using $MAPbI_3$ as an active layer under 808 nm or 650 nm CPL illumination. (a) *J*–*V* characteristics and (b) the corresponding *J*–*t* curve of the CPL detector under 808 nm CPL illumination. (c) *J*–*V* characteristics and (d) the corresponding *J*–*t* curve of the CPL detector under 650 nm CPL illumination.

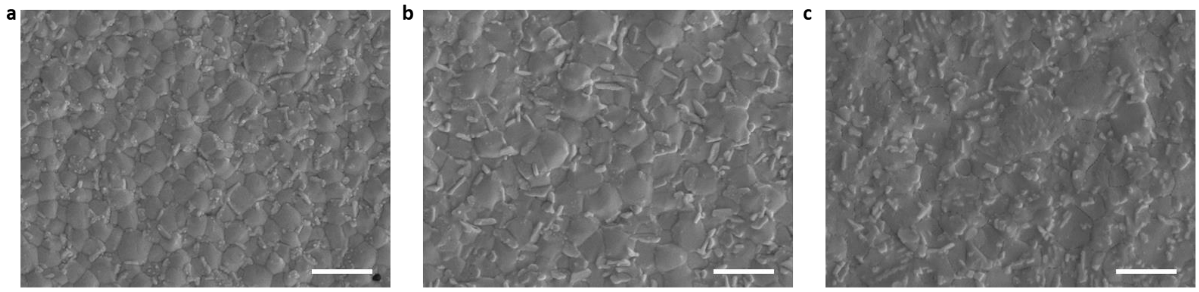

Figure S19. Surface SEM images of perovskite deposited on ITO/AuNP substrate with various perovskite thicknesses: (a) 250 nm, (b), 400 nm, and (c) 800 nm. Scale bars in SEM images indicate 1 μm.

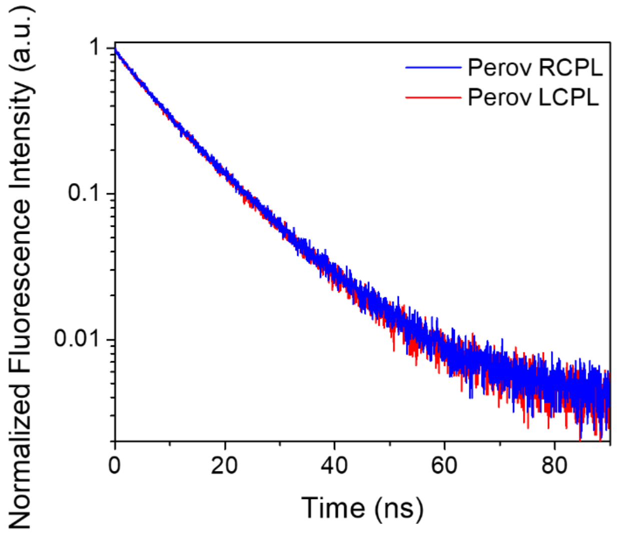

Figure S20. Normalized fluorescence decay profiles for $MAPbI_3$ film without AuNPs under 633 nm CPL excitation.

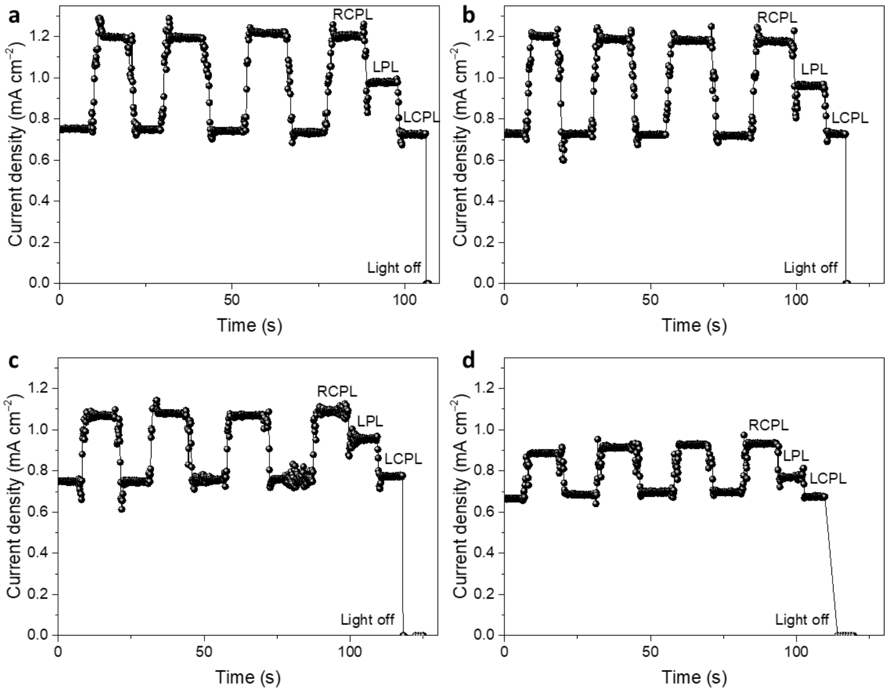

Figure S21. *J*–*t* curves of flexible CPL detectors under 808 nm CPL illumination at various bending radii: (a) 3 cm, (b) 2.5 cm, (c) 2 cm, (d) 1.5 cm.

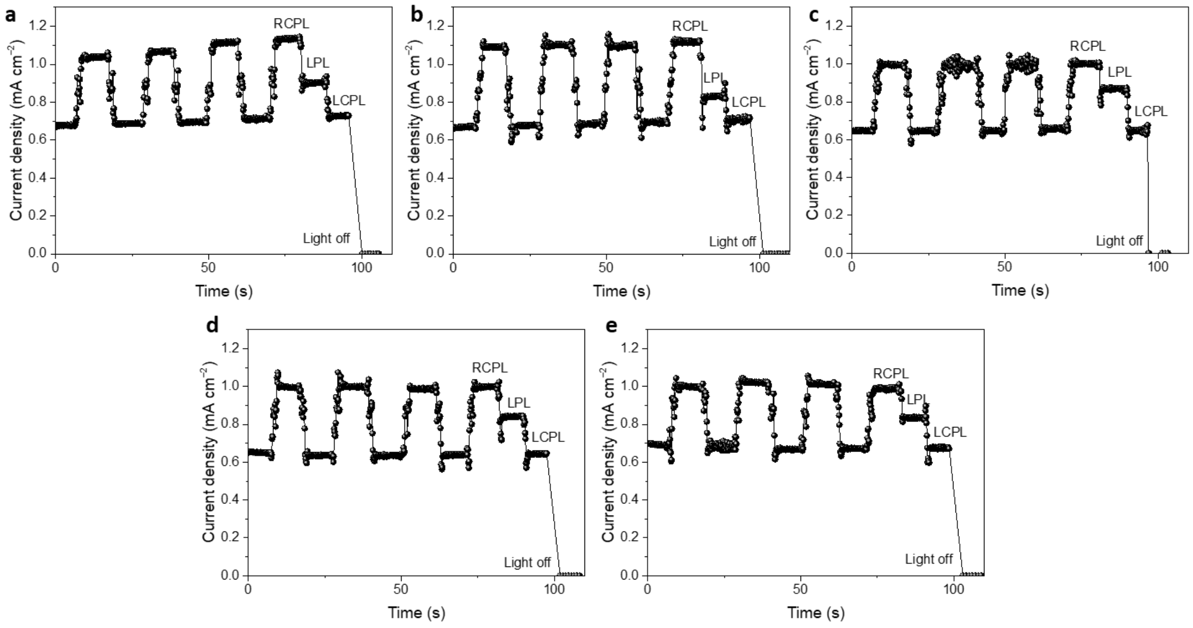

Figure S22. *J–t* curves of flexible CPL detectors under 808 nm CPL illumination after following repetitive bending tests with a fixed bending radius of 2.5 cm: (a) 200 cycles, (b) 400 cycles, (c) 600 cycles, (d) 800 cycles, (e) 1000 cycles.
